# The Fruit Biome: Biofilm Dynamics and Consumer Health Risks with Focus on the Apple (*Malus domestica*) as a Model System

**DOI:** 10.3390/ijms27146247

**Published:** 2026-07-14

**Authors:** Maciej Tankiewicz, Karol Niciejewski, Aleksandra Dydecka, Gracja Topka-Bielecka

**Affiliations:** Department of Environmental Toxicology, Faculty of Health Sciences with the Institute of Maritime and Tropical Medicine, Medical University of Gdańsk, M. Skłodowskiej-Curie 3a Str., 80-210 Gdansk, Poland; maciej.tankiewicz@gumed.edu.pl (M.T.); aleksandra.dydecka@gumed.edu.pl (A.D.); gracja.topka@gumed.edu.pl (G.T.-B.)

**Keywords:** fruit biofilm, apple peel microbiome, postharvest contamination, food safety, foodborne pathogens

## Abstract

Fruit surfaces serve as ecological interfaces that support diverse microbial communities, where biofilm formation by spoilage organisms and human pathogens contributes to postharvest safety concerns. Although fruit-associated microbiota and chemical residues have been widely investigated, the interactions between surface microstructure, residue dynamics, and microbial persistence remain insufficiently integrated. This review synthesizes current knowledge by considering three key processes: temporal succession of microbial communities, structural vulnerability of the fruit surface, and chemically mediated selective pressures. Using apple (*Malus domestica*) as a model system, we examine how structural features such as lenticels and cuticular microdamage interact with pesticide residues to facilitate microbial retention, sequestration, and internalization. Evidence indicates that pesticide residues may act as selective stressors and, in some cases, potential metabolic substrates, thereby enhancing microbial persistence and tolerance to sanitization. These combined factors contribute to the formation of a high-persistence surface environment. Integrating microbiological, chemical, and plant structural perspectives, this review provides a mechanistic basis for the limited effectiveness of conventional decontamination approaches and highlights the need for multidisciplinary postharvest strategies to improve produce safety and shelf life.

## 1. Introduction

The challenge of ensuring food security for a growing global population under conditions of climate change has intensified the development of advanced preservation technologies for fresh produce. Modern postharvest systems, particularly those used for handling fruits such as apples (*Malus domestica*), now enable storage periods extending up to 12 months. Although these technologies are primarily designed to limit water loss and control ripening, they often overlook the complex and dynamic microbial communities inhabiting fruit surfaces [1,2,3].

The fruit surface microbiome constitutes a structured ecological niche in which microorganisms form biofilms—multicellular-like communities embedded within a self-produced extracellular polymeric substance (EPS) matrix. These biofilms can influence fruit physiology and enhance microbial tolerance to environmental stressors, including chemical treatments and desiccation. While they may contribute to natural protective functions, biofilms can also serve as reservoirs for spoilage organisms and human pathogens. Their formation is shaped by factors spanning the entire production chain, from cultivation practices to postharvest handling, and may lead to fruit deterioration, economic losses, and increased risks to consumer health.

Biofilms are inherently complex and composed of diverse microbial populations whose interactions determine their structural stability and functional properties. Their presence on fresh produce facilitates microbial persistence and raises concerns regarding the transmission of pathogenic microorganisms to consumers. Consequently, understanding the dynamics of these communities during storage, as well as the influence of agricultural practices such as pesticide application, is critical for developing effective mitigation strategies. Despite increasing research efforts, important questions remain regarding the evolution of microbial populations during prolonged storage, their impact on the pathogenic potential of biofilms, and the extent to which external interventions modify their structure and composition.

Microbial communities associated with fruit are highly dynamic and undergo significant shifts throughout storage and handling. Environmental parameters such as temperature, humidity, atmospheric composition, and storage duration play key roles in shaping these communities and determining biofilm resilience. Although many microorganisms are neutral or beneficial, others include important foodborne pathogens such as *Salmonella* spp., *Escherichia coli*, and *Listeria monocytogenes*, which pose serious public health risks. The persistence of these organisms within biofilms complicates their removal and increases the likelihood of foodborne infections [1,2,3,4,5].

In addition to physical storage conditions, chemical factors (particularly pesticide residues) can significantly influence biofilm formation and stability. Depending on their chemical properties and concentrations, pesticides may impose selective pressures that alter microbial community composition, promote resistant phenotypes, and induce structural changes in the EPS matrix. Research has demonstrated that exposure to various fungicides and bactericides can modify EPS production and biofilm resilience, either by suppressing or enhancing the persistence of pathogenic microorganisms [6,7]. In field trials, chemical fungicides, such as fenhexamid, significantly reduced microbial richness and diversity in grape biofilm, whereas biological alternatives, specifically *Bacillus subtilis* QST713, largely maintained the ecological balance of the surface community [6]. Further evidence from controlled greenhouse experiments revealed that systemic pesticides, including imidacloprid and metalaxyl, exerted selective pressures that stimulated the growth of specific taxa, such as *Cryptococcus* yeasts and members of the *Enterobacteriaceae* family [7]. These findings indicate that pesticide residues can act as selective stressors or nutrient sources, directly influencing biofilm architecture and potentially facilitating the survival of opportunistic or resistant microorganisms on the fruit surface.

While the ecological principles of biofilm formation and microbial persistence discussed in this review pertain to fresh produce in general, this work utilizes the apple (*Malus domestica*) as the primary model system. This focus is justified by its economic importance and capacity for prolonged postharvest storage, which provides a distinct window for observing microbial succession and biofilm maturation [1,2,3]. Moreover, while the mechanisms of attachment and the health risks posed by pathogens such as *Listeria monocytogenes* or *Salmonella* spp. are broadly relevant across various fruit types [1,2,3,4,5], detailed evidence concerning tissue-specific niches, such as lenticels and the “tear and repair” wax mechanism, is derived specifically from the apple biome [1,4,5].

This review aims to synthesize current knowledge on biofilm formation on fruit surfaces during storage, with particular emphasis on microbial community shifts and their implications for consumer health. Rather than providing a descriptive summary, the work integrates three intersecting dimensions of the topic. First, the temporal successional hypothesis is used to examine how microbial communities evolve during extended storage and how initially low-level contamination may develop into stable, resistant biofilms [1,2,3]. Second, the structural vulnerability hypothesis is considered to assess how the specific microstructure of the fruit surface influences pathogen sequestration, survival, and escape from surface-level sanitization [4,5]. Third, the chemical selective pressure hypothesis is explored to evaluate the extent to which pesticide residues act not only as chemical markers, but also as selective stressors or nutrient sources that modify EPS architecture and promote the emergence of resistant, biofilm-embedded populations [6,7]. By synthesizing these elements, this review establishes a targeted analytical framework to distinguish general microbial ecology from the specific anatomical and chemical factors governing pathogen persistence in the fruit biome, thereby facilitating the development of improved postharvest practices aimed at minimizing the risks associated with biofilm-forming pathogens.

## 2. Methodology of Literature Selection

To ensure a comprehensive and transparent synthesis of current knowledge regarding fruit surface biofilms and consumer health risks, a structured search strategy was employed. The literature search was conducted across several electronic databases, including PubMed, Scopus, Web of Science, and Google Scholar. The search period covered publications from 1990 to June 2026, with a particular emphasis on high-impact research published within the last decade (2015–2026) to reflect the most recent advancements in high-throughput sequencing and molecular microbiology.

The search was performed using specific keywords and Boolean operators, including: (“fruit biofilm” OR “apple peel microbiome”) AND (“postharvest contamination” OR “food safety”) AND (“foodborne pathogens” OR “Listeria monocytogenes” OR “Salmonella”) AND (“mycotoxins” OR “pesticide residues”) AND (“biofilm removal” OR “sanitization”).

Eligibility criteria for including studies in this review were:

1. Peer-reviewed original research articles, comprehensive reviews, and book chapters published in English.
2. Studies focusing specifically on the microbial ecology of fresh produce surfaces, with a primary focus on apples (*Malus domestica*).
3. Research detailing the molecular mechanisms of biofilm formation, persistence, and resistance on biotic surfaces.
4. Studies investigating the interaction between agricultural practices (e.g., pesticide application) and microbial community dynamics.

Exclusion criteria involved:

1. Studies focusing exclusively on clinical biofilm infections unrelated to food matrices.
2. Technical reports or conference abstracts lacking detailed methodologies or peer-review validation.
3. Research concerning processed or cooked foods where the fruit surface biome is no longer intact.

The screening process involved an initial evaluation of titles and abstracts for relevance to the core themes of biofilm dynamics and consumer safety. Subsequently, full-text articles were reviewed to assess their contribution to the mechanistic understanding of the fruit biome. This approach ensured that the selected 126 references represent a balanced and rigorous account of the field, moving beyond a haphazard selection to a focused narrative on the risks and mitigation strategies associated with biofilms formed on fruit.

## 3. The Process of Biofilm Formation on Fruits

Biofilm formation on fruit surfaces is a highly organized, multistage process involving complex microbial communities and tightly regulated molecular mechanisms. A biofilm can be defined as a structured consortium of one or more microbial species embedded within a self-produced matrix composed of organic and inorganic substances. These communities adhere to both biotic and abiotic surfaces and can develop at solid–liquid, liquid–gas, or liquid–liquid interfaces. Importantly, biofilm formation requires only minimal environmental inputs, as the presence of microorganisms, trace nutrients, and limited water availability is sufficient to initiate colonization on virtually any surface, including fruit exteriors [8,9,10,11].

The structural integrity and resilience of biofilms are governed by EPS, which form the matrix embedding microbial cells. This matrix is highly hydrated, with water comprising up to 97% of its total volume, while microorganisms account for only a small fraction. The remaining components include polysaccharides, proteins, lipids, lipopolysaccharides, extracellular DNA (eDNA), and various inorganic compounds, as well as material derived from lysed cells and environmental sources. Together, these elements create a dynamic and protective microenvironment that enhances resistance to environmental stressors, antimicrobial agents, and mechanical removal [8,9,10,11,12].

Within the biofilm, microbial cells are arranged in structured aggregates separated by a network of channels that facilitate the distribution of nutrients, oxygen, and metabolic by-products. While transport processes are most efficient in peripheral regions, cells located in deeper layers rely on diffusion and localized metabolic interactions. This spatial heterogeneity results in physiological diversification, with distinct subpopulations exhibiting specialized metabolic states and stress responses. Such differentiation is further reinforced by signaling networks and gene regulation mechanisms, ultimately giving rise to functionally integrated communities that display characteristics analogous to primitive multicellular systems [9,10,11,13].

Colonization of fruit surfaces by microorganisms is a selective and ecologically driven process shaped by both host and microbial determinants. The fruit peel constitutes a unique interface that simultaneously protects internal tissues from environmental stressors—such as ultraviolet radiation, desiccation, and mechanical damage—and provides a nutrient-rich habitat for microbial growth. Microorganisms capable of successful colonization often possess specific adhesion factors, surface structures (e.g., fimbriae and flagella), and metabolic flexibility that enable them to exploit this niche. Consequently, epiphytic communities form stable and recurrent associations rather than representing incidental contamination [14]. The composition and organization of these communities are strongly influenced by physicochemical characteristics of the fruit surface, including cuticle composition, wax structure, microtopography, and the availability of organic exudates. These factors act as selective filters, determining which microorganisms can adhere, proliferate, and participate in biofilm formation. Additionally, plant-derived compounds, such as polyphenols, may modulate microbial behavior by influencing signaling pathways, stress responses, and efflux systems, further shaping biofilm architecture and persistence [15].

Comparative studies across different fruit species demonstrate that fruit type is a major determinant of microbiome composition and biofilm structure. Fruits such as apples, grapes, strawberries, and peaches harbor distinct microbial consortia with characteristic taxonomic and functional profiles. In apples, the peel supports a particularly structured and metabolically active microbiome that differs markedly from internal tissues, highlighting the importance of tissue-specific niches in plant–microbe interactions. These observations underscore that fruit surfaces function as selective ecological habitats in which microbial community assembly is driven by adaptive processes, interspecies interactions, and finely tuned regulatory mechanisms rather than random colonization [16,17,18,19,20].

### 3.1. Stages of Biofilm Formation

**Figure 1:**
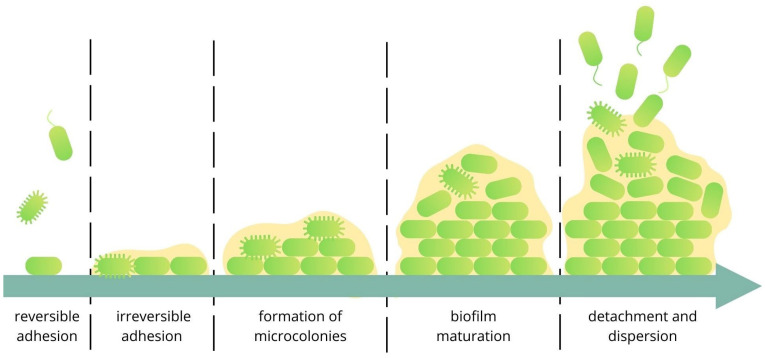
Schematic diagram of biofilm formation based on the five-stage developmental model [8,22].

Biofilm development is a highly coordinated, multistage process governed by both intrinsic microbial properties and the physicochemical characteristics of the colonized surface. It is a dynamic, sequential process in which bacterial cells transition from a planktonic lifestyle to a surface-associated community, accompanied by extensive physiological and phenotypic adaptations [11]. Although the complexity of this process has led to the proposal of several developmental models, a widely accepted framework describes biofilm formation as a five-stage process comprising: (i) reversible adhesion, (ii) irreversible adhesion, (iii) microcolony formation, (iv) maturation, and (v) detachment and dispersion (Figure 1) [8,10,11,21,22,23]. While these stages are conceptually distinct, they represent a continuous and dynamic progression, with each phase contributing to the development of the characteristic structural and functional properties of biofilms [8,9,22].

The initial stage of biofilm formation involves the reversible adhesion of microbial cells to the fruit surface, followed by irreversible attachment and the establishment of stable biofilm development [8]. The subsequent phase involves the formation of microcolonies, in which proliferating cells aggregate into localized clusters embedded within the growing EPS matrix. These structures represent the first level of spatial organization within the biofilm and are characterized by increased cell–cell interactions and metabolic cooperation [11]. Biofilm maturation is marked by the development of a highly organized, three-dimensional architecture accompanied by pronounced physiological differentiation within the matrix. Following initial adhesion, biofilm maturation is driven by EPS production, which forms the structural scaffold of the matrix and promotes long-term persistence on fruit surfaces. EPS enhances cell retention, hydration, and resistance to environmental stress, while also contributing to the stability and resilience of the developing biofilm. The final stage, detachment and dispersion, occurs when the biofilm reaches a critical thickness or undergoes environmental or physiological changes that destabilize its structure [8,9,10,23,24].

#### 3.1.1. Molecular Mechanisms of Biofilm Formation by Bacterial and Fungal Pathogens on Fruit Surfaces

##### Initial Adhesion and Early Biofilm Formation on Fruit Surfaces

Initial adhesion is the key first step in microbial colonization of fruit surfaces, enabling cells to attach to the cuticle or micro damaged tissues and initiate biofilm formation, thereby promoting persistence and increased resistance to environmental stress and sanitization [25]. At the molecular level, adhesion is mediated by specialized surface structures that overcome electrostatic repulsion and establish contact with the substrate. Fimbriae and pili act as filamentous protein appendages facilitating both nonspecific and specific interactions with surface features and conditioning films. At the single-cell level, adhesion is a highly regulated and sequential process involving initial weak physicochemical interactions followed by activation of specific adhesins and irreversible anchoring mechanisms [4]. Curli amyloid fibers encoded by the csg gene cluster form β-sheet-rich extracellular structures that mediate both surface attachment and intercellular cohesion during early biofilm development [26,27]. Type IV pili further contribute by integrating adhesive functions with surface sensing and twitching motility, allowing the transition from reversible to irreversible attachment [26,28,29]. These pili are assembled from PilA pilin subunits and undergo dynamic extension and retraction driven by the antagonistic activity of PilB and PilT ATPases, enabling force generation and surface probing at the nanoscale level [29]. This dynamic behavior allows cells to detect mechanical resistance and translate it into biochemical signals initiating adhesion-specific gene expression programs [28].

Following initial contact, surface-anchored adhesins stabilize adhesion by binding to exposed plant polymers and cuticular components, particularly at microcracks and damaged tissues. Surface sensing triggers rapid intracellular signaling cascades mediated by cyclic di-guanosine monophosphate (cyclic di-GMP), a second messenger that coordinates the repression of motility genes and activation of adhesin and EPS biosynthesis pathways [27,28]. This signaling network represents a key regulatory switch controlling the transition from reversible to irreversible attachment and early biofilm formation [28]. Physicochemical forces, including electrostatic interactions, van der Waals forces, and hydrophobic effects, further modulate adhesion efficiency, with quantitative studies demonstrating that increased hydrophobicity of Salmonella cells significantly enhances adhesion strength and persistence on abiotic surfaces [25]. These physicochemical interactions act synergistically with biological adhesins, forming a multilayered adhesion mechanism at the cell–surface interface [25,27]. Surface roughness and organic conditioning films promote irreversible attachment and microcolony formation [25,27,28].

Structural components of the bacterial envelope play a critical role in this process: lipopolysaccharides (LPS) in Gram-negative pathogens such as *Salmonella* spp. and *Shiga* toxin–producing *Escherichia coli*, and teichoic acids in Gram-positive taxa including *Listeria monocytogenes* and *Bacillus cereus*, regulate cell surface charge and hydrophobicity, thereby strengthening early adhesion and biofilm initiation [30,31]. Flagella-mediated motility indirectly enhances early biofilm formation by enabling active surface exploration and increasing the likelihood of encountering favorable attachment sites such as lenticels, stem scars, and micro damaged tissues. Beyond locomotion, flagella function as mechanosensitive organelles in which mechanical interference with rotation upon surface contact initiates signaling pathways that promote adhesion and biofilm development [28,32]. Motile strains of foodborne pathogens, including *Salmonella enterica* and *E. coli*, exhibit more efficient initial adhesion and microcolony establishment than non-motile counterparts [32]. This coupling of motility, sensing, and adhesion represents an integrated strategy that accelerates surface colonization under dynamic environmental conditions [32].

Early biofilm formation is further driven by the synthesis and spatial organization of EPS, including cellulose, poly-β-1,6-N-acetyl-D-glucosamine (PGA), proteins, and eDNA, which together form a viscoelastic and highly hydrated matrix. At the molecular level, this matrix functions as a diffusion barrier, mechanical scaffold, and biochemical interface that coordinates nutrient retention, cell signaling, and protection against environmental stressors [10]. The multifunctionality of the EPS matrix is a defining feature of early biofilm stability and resilience [30,31].

Beyond bacteria, fungi and yeasts employ analogous but structurally different adhesion mechanisms. Fungal cells express cell wall adhesins, often glycoproteins, that mediate binding to surface substrates, while filamentous fungi and certain yeasts form hyphal or pseudohyphal structures that anchor cells within microscopic surface irregularities, promoting persistent colonization [33,34]. In postharvest fungi, adhesion is closely coupled with the secretion of cell wall-degrading enzymes such as cutinases and polygalacturonases, which enzymatically modify plant surface structures and expose new binding niches at the molecular level [26,35,36]. Ultrastructural analyses indicate that these processes are accompanied by localized cell wall degradation and tissue maceration, facilitating deeper penetration, and stabilization of the fungal structures [37]. Additionally, secondary metabolites such as patulin and ochratoxin A contribute to niche establishment by altering host tissue integrity and influencing microbial competition [26,36]. Filamentous growth in *Rhizopus stolonifer* enables extensive hyphal penetration and mechanical anchoring within micro damaged tissues, reinforcing long-term colonization [37,38]. In parallel, these adhesion- and invasion-associated traits support the formation of fungal biofilm-like communities embedded in an extracellular matrix, which further enhances persistence on fruit surfaces and increases tolerance to postharvest stress [15,20,37]. This matrix-associated lifestyle is particularly relevant in storage conditions, where fungal colonization can become spatially organized and more difficult to remove once established [20,39].

From a postharvest perspective, these mechanisms are highly relevant, as adhesion is a prerequisite for biofilm development in both bacteria and fungi. Interfering with early molecular events—including disruption of curli assembly, inhibition of Type IV pili dynamics, modulation of cyclic di-GMP signaling pathways, or suppression of fungal extracellular enzyme activity and hyphal development—represents a targeted strategy to prevent stable microbial attachment and subsequent biofilm maturation. Strategies that interfere with early attachment—such as reducing hydrophobic interactions or blocking adhesin–substrate binding—can limit microbial settlement and delay biofilm formation, which is considerably easier than removing mature, EPS-protected biofilms [25].

##### Molecular Mechanism of EPS Production and Maintenance in Biofilms on Fruit Surfaces

Following initial adhesion, biofilm maturation on fruit surfaces is driven by the synthesis of EPS, which constitutes the structural backbone of microbial biofilms and enables long-term persistence on fruit cuticles, lenticels, stem scars, and micro damaged tissues. EPS forms a heterogeneous matrix composed primarily of extracellular polysaccharides, proteins and glycoproteins, eDNA, and minor lipid fractions, which embed microbial cells and anchor them to biotic food surfaces [40,41]. EPS production is governed by dedicated biosynthetic gene clusters encoding glycosyltransferases, polymerization and modification enzymes, and export systems responsible for exopolysaccharide assembly and secretion across the cell envelope. These biosynthetic pathways involve specific glycosyltransferases that catalyze the transfer of activated sugar precursors (e.g., UDP-glucose, GDP-mannose) to elongating polysaccharide chains, as well as membrane-associated synthase complexes responsible for polymer assembly and export [42]. EPS biosynthesis is further coordinated by multi-enzyme complexes that ensure spatial coupling between polymerization and translocation across the cell envelope [40]. At the regulatory level, EPS synthesis is tightly coupled to intracellular signaling networks, with c-di-GMP acting as the central second messenger controlling the transition from a motile to a sessile, matrix-producing phenotype. Elevated c-di-GMP concentrations activate transcriptional regulators and directly modulate EPS biosynthetic enzymes, thereby enhancing polysaccharide production while repressing flagellar motility, stabilizing attachment and promoting biofilm accumulation on fruit surfaces [42]. At the genetic level, EPS production is regulated by complex operons and transcriptional regulators that respond dynamically to environmental stimuli, enabling rapid adaptation of the biofilm architecture [42].

Extracellular polysaccharides provide the primary scaffold of the biofilm matrix, conferring mechanical stability, hydration, and resistance to environmental stresses encountered during fruit washing, handling, and storage. Structurally, these polysaccharides form highly hydrated, three-dimensional networks stabilized by hydrogen bonds and ionic interactions, which determine the viscoelastic properties of the biofilm matrix [40]. The physicochemical properties of EPS, including charge density and polymer branching, directly influence adhesion strength, diffusion properties, and resistance to mechanical removal [40,43]. Matrix-associated proteins and glycoproteins further reinforce biofilm cohesion by cross-linking polysaccharide chains and mediating cell–cell interactions, although these components remain analytically challenging to detect in food-associated biofilms. Extracellular DNA additionally contributes to matrix integrity through electrostatic interactions with other EPS components and reduces susceptibility to sanitizing agents commonly applied in postharvest processing [40,41]. It also participates in horizontal gene transfer within biofilms, facilitating the spread of traits associated with persistence and environmental adaptation [41].

Although these mechanisms are best characterized in bacteria, functionally analogous EPS-mediated strategies also operate in fungal and yeast biofilms. In these systems, matrices enriched in glucans, mannans, and glycoproteins similarly support adhesion, structural stability, and persistence on fruit cuticles and within micro damaged tissues [40]. Fungal EPS matrices are dominated by β-1,3-glucans, β-1,6-glucans, and mannoproteins, which form cross-linked networks contributing to mechanical strength and environmental resistance [40]. Dynamic remodeling of these polysaccharides enables fungal biofilms to adapt to changing environmental conditions and stress factors encountered during postharvest handling [43]. In addition, fungal biofilm development is closely associated with morphogenetic transitions, such as yeast-to-hyphae switching, which enhances surface colonization and facilitates deeper penetration into fruit tissues, particularly in damaged or senescent areas [20,35]. These structural transitions are accompanied by coordinated regulation of EPS-related genes and secretion pathways, allowing rapid modulation of matrix composition in response to nutrient availability and environmental stressors [40,42]. Moreover, fungal EPS matrices can interact with host-derived compounds, including cuticular waxes and phenolic substances, further stabilizing attachment and influencing the physicochemical properties of the biofilm microenvironment [39,44]. From a postharvest processing perspective, EPS production represents a major barrier to effective sanitation, as the matrix restricts disinfectant penetration and enhances biofilm resilience. Consequently, strategies targeting EPS biosynthesis, disrupting c-di-GMP signaling, or enzymatically degrading matrix components such as polysaccharides and eDNA are highlighted as promising approaches to destabilize biofilms and reduce microbial contamination on fruits and processing equipment [41,42,43]. Importantly, in fungal systems, additional control strategies may involve targeting cell wall polysaccharide synthesis (e.g., β-glucan synthase inhibitors) or disrupting hyphal integrity, which can indirectly weaken EPS matrix cohesion and improve the efficacy of antifungal treatments [26,43].

##### Efflux Pump-Mediated Biofilm Formation

The increase in bacterial resistance is, among other factors, due to the activity of efflux pumps—transport proteins located in the bacterial cell membrane of both Gram-positive and Gram-negative bacteria. One of their primary functions is to extrude antibacterial compounds, such as antibiotics and the active compounds of disinfectants. Consequently, the presence of efflux pumps can impair the effectiveness of disinfecting products intended for consumers and, in the case of bacterial infections, reduce the efficacy of antibiotic therapy. At the molecular level, efflux pumps function as energy-dependent transport systems driven either by ATP hydrolysis (ABC transporters) or proton-motive force (e.g., RND and MFS systems), enabling active extrusion of structurally diverse substrates [45,46,47]. Efflux pumps also contribute to biofilm formation by modulating adherence, gene regulation (including quorum sensing), and EPS production. Mechanistically, efflux systems can export quorum sensing molecules and other signaling mediators, thereby influencing cell–cell communication and coordinating biofilm-associated gene expression [45]. Bacteria frequently isolated from the surface of fresh fruit, including *E. coli*, *L. monocytogenes*, and *Salmonella* spp., are capable of forming biofilms, a process that is often supported by efflux pump systems [45,46,47,48,49].

Bacterial efflux pumps have been classified into six families, which transport a variety of substances—both endogenous metabolites and compounds introduced into the cytoplasm: ATP-binding cassette (ABC), major facilitator superfamily (MFS), resistance–nodulation–division (RND), small multidrug resistance (SMR), multidrug and toxic compound extrusion (MATE), and proteobacterial antimicrobial compound efflux (PACE). Among Gram-negative bacteria, RND-family efflux systems have the broadest substrate spectrum, actively extruding antibiotics, detergents, disinfectants, and plant-derived phenolic compounds, as exemplified by the AcrAB–TolC pump in *E. coli*, whose expression can be induced by chemical stressors. The AcrAB–TolC system forms a tripartite complex spanning the inner membrane, periplasm, and outer membrane, enabling direct extrusion of substrates into the external environment [47]. The induction of efflux pump systems is regulated by complex stress–response networks and may be influenced by multiple environmental factors. These regulatory networks involve global transcriptional regulators and stress–response pathways that respond to toxic compounds, oxidative stress, and membrane damage [45,47]. Plant-derived polyphenolic compounds, highly abundant in fruit peels, are one example of chemical stressors that can modulate efflux pump expression and contribute to bacterial biofilm formation [45,47,50,51].

Efflux pumps are also present in fungi and are one of the key mechanisms of their resistance to antifungal drugs. Fungal microorganisms present two major groups of efflux pumps: ABC and MFS transporters. ABC transporters are primary active transporters that harness ATP hydrolysis at conserved nucleotide-binding domains (NBDs) to drive substrate translocation across the plasma membrane [52]. In contrast, MFS transporters function as secondary active transporters, utilizing the proton-motive force and electrochemical gradient to drive substrate efflux [52,53]. In fungal pathogens, pleiotropic drug resistance (PDR) ABC transporters have a distinct domain architecture (N–NBD1–TMD1–NBD2–TMD2–C) that differs from mammalian transporters. Furthermore, these fungal pumps contain large, highly conserved extracellular loops (EL3 and EL6) that are important for proper folding and function, and may represent potential targets for selective antifungal therapy [52]. These transporters actively remove antifungal agents from fungal cells, reducing intracellular drug accumulation and enabling survival under fungicide exposure [52,53,54]. Studies have shown that the use of fungicides, such as fludioxonil or azoles, can stimulate the transcriptional activation of efflux pump genes, allowing fungal strains to survive [52,53]. Overexpression is frequently mediated by gain-of-function mutations in fungal-specific Zn2Cys6 transcription factors, such as Mrr1 or ShXDR1, or by promoter rearrangements leading to constitutive efflux pump activation [53]. In *Penicillium expansum*, the primary cause of blue mold in apples, differential expression of ABC and MFS transporters has been linked not only to multidrug resistance but also to increased aggressiveness and mycotoxin (patulin) production [54]. Although MDR strains often exhibit reduced aggressiveness and form smaller lesions on apple fruit compared to wild-type isolates, they may paradoxically produce more patulin. This risk may be further enhanced by exposure to sublethal fungicide concentrations, which can stimulate secondary metabolism [54].

To limit biofilm formation, efflux pump inhibitors (EPIs) can be applied, which block the pumps, thereby, among other effects, inhibiting the active expulsion of antibacterial compounds by bacteria. At the molecular level, EPIs act by disrupting transporter function, inhibiting energy coupling, or blocking substrate-binding sites within efflux systems [52]. Some research indicates that one type of EPI is essential oils—secondary plant metabolites capable of affecting efflux pump activity. Certain essential oil components have been shown to downregulate efflux pump gene expression (e.g., mex systems in *Pseudomonas aeruginosa*), thereby increasing bacterial susceptibility to antimicrobial agents [55]. Studies show that it is also possible to modulate the activity of efflux pumps in fungi by using inhibitors, which can reverse resistance to fungicides and enhance their effectiveness [52,55]. Effective inhibitors of fungal efflux pumps include synthetic D-octapeptide derivatives, such as RC21v3, which target the unique extracellular loops (ELs) of the Cdr1 pump. Milbemycins and enniatins can also chemosensitize resistant strains to azoles by interfering with ATPase activity or the substrate channel [52].

##### Quorum Sensing in Biofilm Development

Quorum sensing (QS) is an intercellular communication mechanism, primarily in bacterial cells, that regulates the production of virulence factors, including biofilm formation. At the molecular level, QS relies on the synthesis, release, detection, and signal-dependent transcriptional response to autoinducers, which coordinate gene expression across the bacterial population [56,57]. In this process, autoinducers (small signaling molecules) mediate bacterial communication and gene expression through quorum sensing. Bacteria release autoinducers that accumulate as the population grows, leading to the activation of genes responsible for the production of biofilm matrix components, adhesion to the fruit surface, and toxin production. Once a threshold concentration is reached, autoinducers bind to specific receptors or transcriptional regulators, triggering signal transduction cascades that activate QS-controlled genes [57,58]. Bacterial QS systems are mainly classified by the type of autoinducer, with three main types distinguished: the acyl-homoserine lactone (AHL) system in Gram-negative bacteria, the autoinducing peptide (AIP) system in Gram-positive bacteria, and the autoinducer-2 (AI-2) system in both Gram-negative and Gram-positive bacteria [56,57,58]. In Gram-negative bacteria, AHL molecules freely diffuse across membranes and bind to LuxR-type regulators, forming active transcriptional complexes [57]. In Gram-positive bacteria, AIP signals are detected by membrane-bound two-component systems consisting of histidine kinases and response regulators [56,57].

*E. coli*, which are commonly found on the surfaces of fruits, primarily exhibit QS through the autoinducer-2 (AI-2) system. González Barrios et al. demonstrated that AI-2 directly stimulates biofilm formation in *E. coli*, increasing its mass (up to 30-fold), thickness, and altering its architecture, confirming the key role of quorum sensing [59]. Mechanistically, AI-2 regulates biofilm formation through the MqsR regulator, which links quorum sensing with motility control and stress–response pathways. In turn, *Salmonella* spp., which also frequently occur on fruit surfaces and are capable of forming biofilms, possess quorum sensing systems mediated by three autoinducers (AI-1, AI-2, and AI-3). In *Salmonella Enteritidis*, AHL-based QS systems have been shown to enhance biofilm formation under specific environmental conditions, including anaerobic environments [60]. Some studies have shown that mutated strains, lacking AI-2, exhibit reduced expression of genes involved in biofilm formation, resulting in decreased biofilm-forming ability [60,61]. Transcriptomic analyses indicate that the luxS/AI-2 system regulates genes associated with metabolism, motility, and stress adaptation, all of which contribute to biofilm development [61].

QS is also among the most extensively studied mechanisms of cell–cell communication in fungi. This process plays a crucial role, particularly in the yeast-to-hyphae transition and in interspecific interactions, primarily with bacteria [62]. In fungal systems, QS molecules such as farnesol and tyrosol regulate morphogenesis, biofilm formation, and virulence through modulation of signaling pathways controlling cell differentiation. In *Candida albicans*, farnesol functions as a quorum-sensing molecule that inhibits the Ras1-cAMP-PKA pathway and suppresses hyphal initiation, whereas tyrosol stimulates filamentation and biofilm maturation. In *Saccharomyces cerevisiae*, aromatic alcohols, including phenylethanol and tryptophol, link nitrogen depletion to filamentous growth. In *Cryptococcus neoformans*, the quorum-sensing peptide Qsp1 regulates virulence-associated traits. Quorum sensing also contributes to immune evasion through PAMP masking, including α-1,3-glucan production in *Histoplasma capsulatum* and β-glucan masking in *Candida albicans* [62].

QS inhibitors are a promising strategy to control biofilm formation and virulence in bacteria and fungi. In the case of bacteria, QS inhibitors may include natural compounds such as organic acids, which can reduce biofilm formation and virulence [63]. At the molecular level, these compounds interfere with QS by inhibiting signal synthesis, degrading autoinducers, or blocking receptor binding, thereby disrupting coordinated gene expression. Such interference leads to attenuation of virulence factor production and impaired biofilm maturation, making QS a key target for antimicrobial strategies [56,63].

### 3.2. Determinants of the Formation of Microbial Biofilms on Fruit Peel

Environmental conditions are among the principal determinants of biofilm formation, though their impact is highly species-specific and qualified by the colonized matrix and nutrient availability. Temperature is particularly important, as most bacteria form biofilms efficiently within the range of 20 °C to 37 °C. For instance, *Aeromonas hydrophila* exhibits optimal biofilm formation and quorum sensing at 20–25 °C, whereas selected strains of *Klebsiella pneumoniae* and *Salmonella* demonstrate maximal activity at 22–30 °C or 40 °C. However, the relative importance of environmental factors may change over the course of incubation. Multivariate analyses indicate that temperature is the primary determinant at 25 °C, whereas incubation time becomes more influential at 37 °C for many bacterial isolates [64]. Pathogens such as *Salmonella enterica* and *Escherichia coli* may persist and form biofilms at both ambient and cold storage temperatures, whereas psychrotrophic bacteria, including *Pseudomonas* spp., remain metabolically active at low temperatures. Humidity and water activity further support biofilm development by facilitating microbial survival, motility, and attachment, while fluctuations in moisture may select for more resilient and drought-tolerant communities [64,65,66].

Nutrient availability also exerts a substantial influence on biofilm formation. Although nutrient-rich conditions generally favor microbial growth, nutrient limitation can paradoxically stimulate biofilm production as part of an adaptive stress response. Certain compounds, including glucose, NaCl, and phosphorus, may enhance adhesion and increase biofilm robustness by modifying cell surface properties. Specifically, phosphorus has been shown to increase cell surface hydrophobicity, thereby promoting bacterial attachment. In addition, the biochemical composition of fruit peels, including sugars, organic acids, and micronutrients, creates localized microenvironments that support microbial colonization [64].

Microbial factors, including initial population density, growth phase, and interspecies interactions, are equally important. A higher microbial load increases the likelihood of cell aggregation and rapid biofilm establishment. Cells in the logarithmic growth phase tend to adhere more efficiently than those in the stationary phase. Moreover, mixed-species biofilms often exhibit enhanced stability and resistance due to synergistic interactions between microorganisms [64,67].

The physical and chemical characteristics of fruit peels strongly influence microbial attachment and subsequent biofilm development. Surface roughness, hydrophobicity, wax composition, and the presence of polysaccharides all affect the ability of microorganisms to adhere and persist. Compared with abiotic surfaces, such as stainless steel or polystyrene, biotic matrices can support the development of more complex three-dimensional biofilm structures [66]. Microbial surface structures, including curli fimbriae and cellulose, facilitate attachment, while the EPS matrix-composed of polysaccharides, proteins, lipopolysaccharides, and extracellular DNA-anchors the biofilm to the fruit surface and protects embedded cells from environmental challenges, including antimicrobial treatments [68,69].

Biofilm formation is also closely associated with stress adaptation and cell-to-cell communication. Environmental stressors such as nutrient limitation, temperature fluctuations, and exposure to sanitizing agents can induce biofilm formation. In parallel, quorum sensing systems regulate the expression of genes involved in adhesion, EPS synthesis, and biofilm maturation, thereby enabling coordinated community behavior and structural organization [64,68,69,70]. Additional external factors, including pesticide application, climate change, and postharvest handling, further shape biofilm dynamics. Pesticides may exert dual effects: some compounds display direct antimicrobial activity, whereas others can be metabolized by microorganisms and used as nutrient sources, thereby promoting biofilm growth. Repeated exposure to these agents may also select for resistant, biofilm-forming strains. Climate change, through rising temperatures and altered humidity patterns, may further favor biofilm development and resilience by increasing microbial metabolic activity and selecting for stress-tolerant populations. Importantly, biofilm formation is not limited to the field but continues throughout storage and distribution. Microorganisms can develop biofilms on fruit surfaces under both refrigerated and ambient conditions, allowing them to survive from harvest to retail. These biofilms protect microbial communities against cold, desiccation, and sanitizing agents, thereby increasing the persistence of pathogens and the risk of foodborne outbreaks [71].

In summary, microbial biofilm formation on fruit peels results from the combined effects of environmental factors, microbial traits, and surface characteristics. Temperature, humidity, nutrient availability, microbial interactions, surface properties, pesticide exposure, climate change, and storage conditions collectively determine the extent and persistence of these structures. A comprehensive understanding of these determinants is essential for developing effective strategies to control biofilm formation and improve the safety of fresh produce.

### 3.3. Sources of Food Spoilage Microorganisms

**Figure 2:**
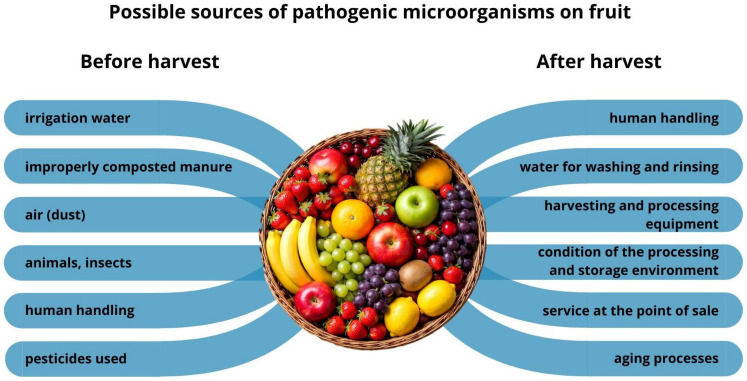
Possible sources of pathogenic microorganisms on fruit.

Microorganisms are present at every stage of the fruit production chain and may enter food from both natural and anthropogenic sources. Contamination can occur in the orchard, during harvest, in packing and storage facilities, during transport, and at the retail stage. The possible sources of pathogenic microorganisms on fruits are summarized in Figure 2 [72,73].

From a biological perspective, fruit contamination reflects the interaction between the production environment and the microbial ecology of the crop itself. Soil, irrigation water, air, insects, animals, and plant-associated microbiota may all serve as reservoirs of spoilage organisms and potential pathogens. Among these, soil and water are particularly important because they can repeatedly contaminate the fruit surface and promote the persistence of microorganisms under favorable moisture and temperature conditions [72,73].

Anthropogenic sources are equally relevant. Agricultural workers, harvesting tools, crates, packing lines, storage rooms, and transport equipment may contribute to cross-contamination when hygiene practices are inadequate. Even minor mechanical damage to the peel can increase susceptibility to colonization by exposing internal tissues and creating microhabitats that support microbial attachment and biofilm development. In this way, postharvest handling may amplify contamination that initially arose in the field. A key issue is that contamination is not limited to the presence of microorganisms alone, but also depends on their ability to survive and proliferate on fruit surfaces. Once established, some spoilage organisms may persist as part of mixed microbial communities, while others may exploit favorable storage conditions to grow and outcompete the native microbiota. This is especially important in fruits consumed raw or with minimal processing, where there is no terminal kill step to eliminate microorganisms before consumption.

In summary, the sources of food spoilage microorganisms are diverse, interconnected, and difficult to eliminate once contamination has occurred. Their impact depends not only on the origin of the organisms, but also on storage conditions, surface damage, and the capacity of microorganisms to persist in biofilms. A comprehensive understanding of these routes is essential for improving fruit safety and for designing preventive measures that reduce contamination throughout the production chain [72,73].

## 4. Microbial Communities on Fruit Surfaces

Fruits are generally regarded as safer than many other unprocessed foods, yet they are by no means sterile. Their surfaces harbor diverse epiphytic microorganisms, while internal tissues contain endophytic taxa, together forming a functional microbiome that interacts with the fruit throughout development, harvest, and storage. These fruit-associated communities typically comprise a broad range of bacteria and fungi that may influence postharvest physiology, fruit quality, and natural resistance to pathogens [15,17].

High-resolution studies of the apple microbiome have shown that the surface community is both taxonomically rich and dynamically structured. Its composition can vary substantially depending on cultivar, orchard management practices, storage conditions, developmental stage, postharvest interval, and the microbial composition of the surrounding air. In addition, global analyses have demonstrated pronounced biogeographical patterns, indicating that microbial assemblages on apples differ markedly between geographic regions and production systems. Recent studies also suggest that orchard management can influence not only community composition but also functional potential, including traits related to plant defense and antimicrobial activity [15,74,75].

Beyond environmental and management-related factors, intrinsic fruit characteristics strongly modulate microbial colonization. Surface pH, cuticle structure, wax composition, moisture availability, and microtopography all influence the ability of microorganisms to attach, persist, and form stable communities. These parameters vary among fruit species and even among tissues of the same fruit, which helps explain why produce types differ substantially in their microbial load and taxonomic composition. The fruit peel therefore acts as a selective ecological niche rather than a passive surface, favoring microorganisms adapted to local physicochemical constraints [15,17,44,76,77,78,79].

Microbial diversity is also shaped by hygiene standards during harvesting, processing, storage, and distribution. Improved sanitation and careful handling generally reduce the abundance and diversity of surface microbiota, whereas inadequate hygiene increases the likelihood of cross-contamination and the persistence of undesirable taxa. This is especially relevant for fruits that are consumed raw, since the surface community may directly influence consumer exposure to spoilage organisms and opportunistic pathogens [15,17,44,74,75,76,77,78,79].

Overall, fruit surfaces support complex and highly variable microbial communities whose structure reflects the combined effects of host traits, environmental exposure, production practices, and storage conditions. In apples, in particular, the microbiome appears to be shaped by cultivar and management practices as well as geography, emphasizing that microbial assembly is both context dependent and biologically meaningful. Understanding these communities is therefore essential for interpreting fruit spoilage dynamics and for designing postharvest strategies that preserve quality while limiting health risks [74,75,76].

### 4.1. Bacterial Communities Colonizing Fruit Surfaces

The composition of microbial communities inhabiting fruit surfaces is strongly influenced by the type of fruit, with different produce harboring distinct bacterial and fungal assemblages. Apples, grapes, strawberries, and peaches, for example, support large and taxonomically diverse bacterial populations dominated by members of the Actinomycetota, Bacteroidota, Bacillota, and Pseudomonadota. Within apples specifically, Alphaproteobacteria represent the most abundant bacterial class, and Sphingomonadaceae have been identified as the dominant family associated with the apple surface. In contrast, strawberries exhibit a high prevalence of Enterobacteriaceae, whereas apples, peaches, and grapes contain only very low proportions of this family. More recent work confirms that the apple peel hosts a highly structured and tissue-specific microbiome distinct from that of internal tissues, dominated primarily by Pseudomonadota but also containing Actinomycetota, Bacillota Bacteroidota, and a substantial fraction of unclassified bacterial lineages. At the family level, peel-associated communities comprise characteristic plant epiphytes such as Enterobacteriaceae, Pseudomonadaceae, Sphingomonadaceae, Methylobacteriaceae, Microbacteriaceae, and Methylocystaceae [15,16,17,18,19,20,39].

In general, fruit surfaces are colonized predominantly by nonpathogenic epiphytic microorganisms occurring as vegetative cells or spores. Although the fresh fruit peel represents a relatively inhospitable habitat, both Gram-positive and Gram-negative bacteria are consistently recovered from these surfaces. Gram-negative populations commonly include members of Pseudomonadaceae (*Pseudomonas*, *Xanthomonas*), Enterobacteriaceae (*Enterobacter*, *Aerobacter cloacae*, *Erwinia*), and Achromobacteriaceae (*Flavobacterium*). Gram-positive genera frequently detected include *Bacillus* and *Lactobacillus* (Bacillaceae), as well as coccoid taxa within Micrococcaceae (*Micrococcus*), Staphylococcaceae (*Staphylococcus*), and Streptococcaceae (*Streptococcus equines*, *S. salivarius*, *S. faecalis*, *S. mitis*) and Sarcinaceae. Additional investigations of raw fruits have reported the presence of *Klebsiella* spp., *Citrobacter* spp., *Enterobacter* spp., *Escherichia coli* and *Staphylococcus aureus* [44,80]. All bacterial taxa identified on the fruit peel are summarized in Table 1.

### 4.2. Fungal and Yeast Communities Colonizing Fruit Surfaces

Fungal communities similarly exhibit strong tissue specificity and high diversity on fruit surfaces. Two major fungal phyla dominate the peel environment: Ascomycota and Basidiomycota, with Ascomycota representing the majority across samples. Genus-level taxonomic resolution reveals several fungal taxa characteristic of apple surfaces, including *Aureobasidium*, *Pseudomicrostroma*, *Penicillium*, *Ramularia*, *Cladosporium*, *Alternaria*, *Paraconiothyrium*, and *Golubevia*. Notably, *Penicillium*, along with an unidentified Ascomycota genus, shows markedly higher abundance in the peel compared with internal tissues, suggesting preferential colonization of the fruit surface by these fungi. The general fungal microflora of fruits is equally diverse, encompassing both filamentous fungi and yeasts, as summarized in Table 2. Common molds include *Rhizopus*, *Aspergillus*, *Penicillium*, and *Wallemia*, whereas the yeast community comprises genera such as *Saccharomyces*, *Zygosaccharomyces*, *Hanseniaspora*, *Candida*, *Debaryomyces*, and *Pichia*. Together, these bacterial and fungal taxa constitute a complex, multilayered epiphytic microbiome shaped by fruit type, tissue structure, and surface physicochemical properties [39,44].

### 4.3. Plant Pathogens and Postharvest Spoilage Organisms

Although fruits are typically colonized by diverse nonpathogenic microorganisms, they may also harbor important human pathogens, including *Staphylococcus aureus*, *Listeria monocytogenes*, *Escherichia coli*, and *Salmonella* spp. This is of particular concern because fresh fruits are frequently consumed raw, allowing contaminated produce to serve as a vehicle for foodborne outbreaks. In addition to human pathogens, fruit quality and yield are substantially affected by plant pathogens and postharvest spoilage organisms. Among the most important fungal agents are *Penicillium expansum* and *Botrytis cinerea*, which readily infect wounds on fruit surfaces. If such infections are not eliminated before storage, these fungi can rapidly degrade tissue, produce visible lesions, and facilitate cross-contamination between neighboring fruits. Their impact is especially pronounced in apples, pears, and other pectin-rich fruits, where tissue breakdown may proceed quickly under favorable storage conditions. Likewise, bacterial pathogens such as *Erwinia carotovora* subsp. carotovora (syn. *Pectobacterium carotovora*) are highly efficient causes of soft rot in a range of vegetables and some fruits, with the capacity to attack tissues both before and after harvest [81,82,83,84,85].

Beyond these major taxa, fruit spoilage is commonly associated with a broad spectrum of fungal genera, including *Alternaria*, *Aspergillus*, *Botrytis*, *Colletotrichum*, *Fusarium*, *Monilinia*, *Penicillium*, and *Rhizopus*. Similarly, bacterial spoilage is associated with genera such as *Clostridium*, *Pectobacterium* (formerly *Erwinia carotovora*), *Clavibacter*, *Streptomyces*, and *Xanthomonas*, whereas microbial contamination of fruits may also involve foodborne pathogens including *Listeria monocytogenes*, *Salmonella* spp., and *Escherichia coli*. Collectively, these organisms account for much of the microbiological deterioration observed in fruit products and represent an important source of risk for consumers [81,82,83].

The pathogenic and spoilage potential of these microorganisms is strongly influenced by fruit damage, storage conditions, and the ability of the organisms to persist on the peel or within wounds. Once established, they may spread locally within the fruit or disseminate to adjacent produce, particularly when biofilm formation and high humidity favor survival and colonization. For this reason, rigorous sorting, handling, and storage practices are essential to reduce microbial load, limit the spread of infection, and preserve fruit quality throughout the supply chain [81,82,83,84,85].

## 5. The Apple Surface Microbiome

**Figure 3:**
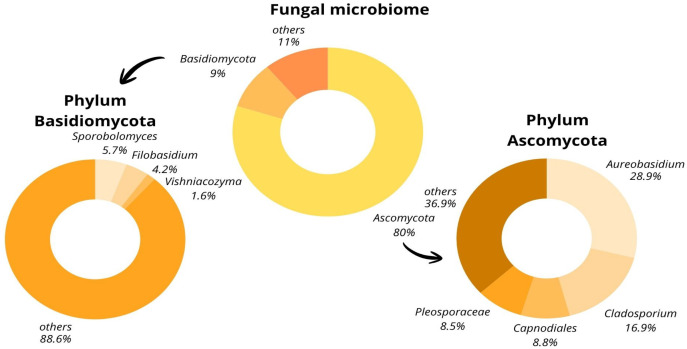
Relative composition of the fungal microbiome associated with apple peel at the phylum and genus levels. The donut charts show the percentage contribution of the main detected fungal taxa, calculated from sequencing-based relative read abundance; “others” combines taxa below the displayed level of resolution [15].

**Figure 4:**
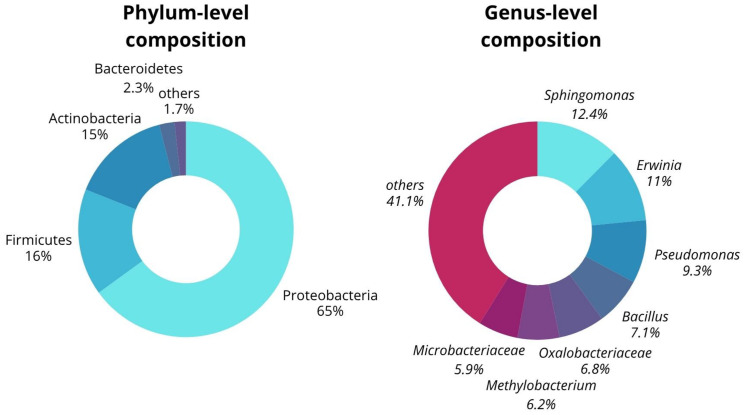
Relative composition of the bacterial microbiome associated with apple peel at the phylum and genus levels. The donut charts show the percentage contribution of the main detected bacterial taxa, calculated from sequencing-based relative read abundance; “others” combines all remaining taxa not listed individually [15].

Apples are among the most widely consumed fruits globally; consequently, the remainder of this review focuses on this fruit. In a study by Abdelfattah et al. investigating the diversity of microorganisms associated with Royal Gala apples, the apple microbiota was reported to be dominated by fungi, as illustrated in Figure 3 [15]. The bacterial microbiome is additionally illustrated in Figure 4 [15].

The “Royal Gala” apple variety was selected for this study because it is one of the most economically important varieties grown in all regions of the world. This allowed the authors to test the hypothesis of a “core” microbiome independent of the location of cultivation [15]. Fruits were harvested at commercial maturity using standard ripeness indices. The study covered four main regions (North America, South America, Europe, and the Middle East), including 21 locations (orchards) in eight countries: the USA, Canada, Uruguay, Italy, Spain, Switzerland, Israel, and Turkey [15].

Eight non-adjacent trees were selected from each orchard. Five fruits were harvested from each tree, taken from different sides of the canopy (around the tree’s circumference). A total of 840 apples were harvested for the study (21 locations × 8 trees × 5 fruits). Fruit from each tree was pooled to create one biological replicate (a total of eight replicates per orchard). Three tissue types (peel, peduncle region, and calyx) were collected from each apple, which, after pooling and processing, resulted in a total of 505 samples for molecular analysis [15]. To avoid errors resulting from different library preparation methods, all extracted DNA samples from different countries were sent to a single center (USDA-ARS in the USA) for sequencing [15].

The authors calculated the percentages of microbial groups based on amplicon sequencing analysis (16S rRNA for bacteria and ITS2 for fungi) using the following methodology.

- Data processing and taxonomic identification: After removing low-quality sequences and sequences of plant origin (chloroplasts and mitochondria), reads were assigned to amplicon sequence variants (ASVs) using the DADA2 pipeline in the Qiime2 environment. Taxonomic assignment was performed using the Greengenes (for bacteria) and UNITE (for fungi) databases at a similarity threshold of 97%.
- Data Normalization (CSS): To enable comparisons between samples and calculate percentages of community composition, the authors used the Cumulative Sum Scaling (CSS) method from the MetagenomeSeq package. This normalization was performed on the unrarefied ASV table.
- Relative Abundance Calculation: Based on the normalized data, the dominant taxa were determined.
- Co-occurrence Analysis: To detect interactions between groups, a Spearman Rank correlation matrix was created, limiting the correlation to genera whose normalized relative abundance exceeded 0.1% in at least one sample.

By using CSS normalization, the researchers were able to precisely determine which genera and families dominate the microbiome of “Royal Gala” apples globally [15].

This analysis enabled the identification of the core microbiome of Royal Gala apples, defined as taxa detected in at least 75% of the samples. This comprised six fungal genera—*Aureobasidium*, *Cladosporium*, *Alternaria*, *Filobasidium*, *Vishniacozyma*, and *Sporobolomyces*—as well as two bacterial genera, *Sphingomonas* and *Methylobacterium*. While no bacterial genera were dominant in 90% of the samples, the fungal genera *Aureobasidium* and *Cladosporium* were found in as many as 96% of the samples. Furthermore, it was shown that the fungal communities were more variable than the bacterial communities in terms of diversity and abundance [15]. Importantly, subsequent studies demonstrated that apple-associated microbial communities vary substantially depending on cultivar, plant organ, orchard management, and geographic origin. In addition to “Royal Gala” apples, microbial profiling has also been performed for commercially important cultivars including Fuji, Golden Delicious, Braeburn, McIntosh, and Granny Smith. A large-scale comparative study by Longa et al. showed that fruits, flowers, bark, and leaves of Gala, Fuji, and Golden Delicious apples harbor distinct fungal and bacterial communities, with Pseudomonadota, Actinomycetota, Bacillota, and Bacteroidota representing the dominant bacterial phyla across cultivars and developmental stages [75]. Similarly, studies conducted on multiple apple cultivars, including Gala, Fuji, and Golden Delicious, demonstrated that microbial community composition is strongly influenced by plant organ and environmental conditions, although cultivar genotype may also contribute to microbiome variation depending on tissue type and experimental system [75]. Furthermore, global-scale analyses confirmed that the composition of fungal and bacterial microbiota differs significantly among apples originating from different geographic regions, indicating that microbiome assembly is shaped by both host genotype and environmental conditions [15].

**Figure 5:**
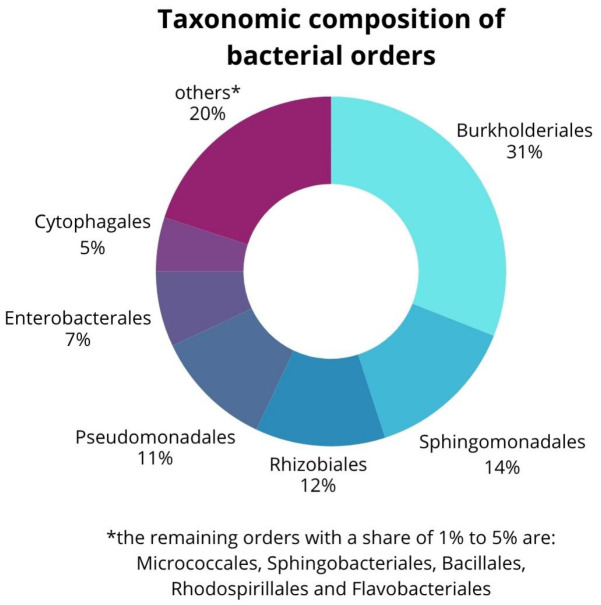
Bacterial microbiome on the surface of an apple [19].

In another study, researchers analyzed fruit from two different cultivation systems: organic and conventional. Microbiome analysis focused exclusively on “Arlet” apples and revealed that the fruit surface is a unique environment characterized by the lowest bacterial counts but also a very high species diversity. Sequencing 16S rRNA gene amplicons on the fruit surface allowed the identification of the bacterial orders shown in Figure 5 [19].

Additionally, the study revealed significant differences in the occurrence of these orders depending on orchard management:

- The order Cytophagales was significantly more abundant in organic apples. The orders Deinococcus-Thermus and Saccharibacteria were also present.
- The orders Burkholderiales, Pseudomonadales, Enterobacteriales, and Flavobacteriales dominated in conventional apples.

The authors paid particular attention to the order Enterobacteriales, as signatures of taxa that may affect health, such as Escherichia-Shigella, which were detected in apples from conventional cultivation systems [19]. In another study of the epiphytic surface microbiome of “Golden Delicious” apples, conducted using shotgun metagenome sequencing, microorganism groups and their percentages were identified [74]. The percentages of microorganisms were calculated based on the taxonomic annotation of the metagene catalog, which was created by assembling raw sequencing reads into contigs and predicting protein-coding open reading frames (ORFs). Taxonomy was assigned to approximately 69% of the identified genes using the LCA algorithm, and count tables were then generated by mapping all sequence libraries to these genes using BWA software (version 0.7.19-r1273). The final percentage of each group was calculated as the ratio of the number of genes (accessions) assigned to a specific taxonomic identifier to the total number of all genes with taxonomic annotations. Based on this, the researchers determined a general domain division, assigning approximately 61% of the annotated genes to bacteria and archaea and 14% to eukaryotes (primarily fungi), and then calculated the detailed phylum proportions within these groups [74].

**Figure 6:**
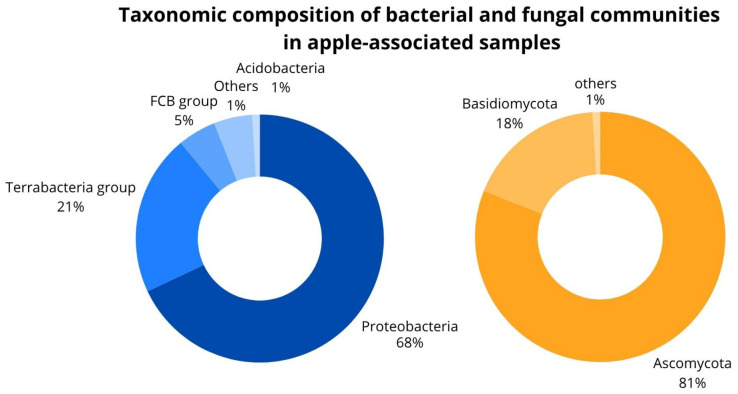
Bacterial and fungal microbiome on the surface of a “Golden Delicious” apple. The figure summarizes major bacterial and fungal taxonomic groups reported in the source study [74].

The proportions of individual phyla within the bacterial and fungal groups are presented in Figure 6 [74].

The authors point out that an important aspect of this study is that the orchard management system (organic vs. conventional) drastically alters the functional profile of the microbiome, even if the taxonomic compositions in different geographic locations show similarities. The microbiome of apples from the organic orchard was significantly enriched in metabolic pathways related to plant defense mechanisms, such as alkaloid and terpenoid biosynthesis, which the authors attribute to the fruit’s greater exposure to pests and pathogens in an environment free of synthetic pesticides. In contrast, conventional orchards demonstrated higher alpha diversity and a microbiome specialized in antibiotic biosynthesis and the metabolism of sulfur-containing amino acids, which may be a direct response to the chemical plant protection programs used. Using innovative metabolic network modeling, researchers were able to link specific groups of microorganisms to their ecological roles, demonstrating, for example, the crucial role of the order Pseudomonadales in terpenoid production in organic orchards and the ability of the pathogenic genus Alternaria to degrade specific aromatic compounds released by apples during ripening [74].

The studies summarized above describe the microbiota of whole fruits rather than individual anatomical compartments and therefore do not fully capture the spatial heterogeneity of the apple microbiome. In a study focused specifically on apple peel, yeasts belonging to the genera *Aureobasidium*, *Metschnikowia* and *Rhodotorula* accounted for the largest proportion of detected microorganisms, whereas potentially pathogenic fungi, including *Penicillium*, *Aspergillus* and *Alternaria*, were present only in low abundance. Bacterial representation was comparatively limited, with *Cronobacter*, *Sphingomonas*, *Methylobacterium*, and *Hymenobacter* among the most frequently detected genera. To obtain a more comprehensive view of community composition, metabarcoding analysis was subsequently applied. This approach, based on the sequencing of short DNA barcode regions, enables simultaneous detection of multiple taxa within a single sample and provides higher taxonomic resolution than conventional methods. Using this strategy, the fungal microbiome of apple peel was shown to be dominated by *Cladosporium*, *Aureobasidium*, *Didymella* and *Vishniacozyma*, while the bacterial community was again mainly represented by *Cronobacter*, *Sphingomonas*, *Methylobacterium* and *Hymenobacter* [15,86]. Additional studies performed on different apple cultivars further support the existence of cultivar-dependent variation in microbial composition. Comparative analyses involving Braeburn, Fuji, Gala, Golden Delicious, Granny Smith, McIntosh, and Red Delicious apples demonstrated substantial differences in fruit biochemical composition, including polyphenol and fiber content, which are recognized determinants of microbial colonization and persistence on fruit surfaces. Granny Smith apples, in particular, were characterized by elevated levels of non-digestible compounds and phenolics, factors that may contribute to selective enrichment of specific microbial taxa. Collectively, these observations indicate that the apple microbiome should not be interpreted as uniform across cultivars, but rather as a dynamic ecological system shaped by cultivar-specific physicochemical traits, orchard environment, and postharvest conditions [87].

Overall, findings from studies performed across multiple apple cultivars and production systems indicate that the apple peel constitutes a distinct microbial niche characterized by a dominant fungal fraction and a comparatively less abundant bacterial community. However, the relative abundance of specific taxa differs among cultivars as well as between orchards and geographic regions. The recurrent presence of epiphytic yeasts together with opportunistic spoilage-associated fungi suggests that the peel is not merely a passive surface, but rather a selective ecological interface involved in microbial assembly and persistence. From a food safety perspective, this supports the view that the apple surface may serve as a reservoir of spoilage organisms and potential contaminants, reinforcing the need for postharvest strategies that account for cultivar-specific and environment-dependent microbiota variation [75,86,87]. Fruits are highly perishable commodities, particularly after harvest, because their tissues remain biologically active and provide a rich source of nutrients for microbial growth [88]. As a result, they create favorable conditions for the development of a wide range of spoilage organisms and opportunistic pathogens. Apples, in particular, are highly susceptible to postharvest fungal infection. Many microorganisms responsible for fruit spoilage are also capable of producing toxic metabolites that may pose a risk to human health if contaminated fruit is consumed. On a molecular level, mycotoxins such as patulin—primarily synthesized by *Penicillium expansum*—exert toxicity through their high affinity for sulfhydryl groups, leading to the inhibition of essential enzymes like ATPase and RNA polymerase [72,89,90,91]. The outer peel represents the first natural barrier against microbial invasion. Under normal conditions, fruits are harvested intact and visually undamaged; however, contamination may occur during cultivation, harvesting, transport, storage, and distribution. Once the peel is colonized by spoilage or pathogenic microorganisms, the surface may serve as a reservoir from which microbes persist and spread. Mechanical injury, such as bruising, cuts, or tearing of the skin, further increases this risk by exposing nutrient-rich internal tissues and providing direct access for microorganisms to proliferate rapidly [72,90].

In this context, peel-associated microbiota should be viewed not only as a marker of surface contamination but also as a key determinant of postharvest fruit deterioration. Recent high-throughput metabarcoding studies utilizing fungal ITS and bacterial 16S rRNA sequencing have identified that apple peels harbor distinct microbial communities often dominated by fungal genera such as *Cladosporium*, *Aureobasidium*, and *Vishniacozyma*, alongside bacterial taxa like *Sphingomonas* and *Methylobacterium*. The diversity of these communities is influenced by abiotic factors, although the effect of controlled atmosphere (CA) storage on microbiome diversity appears to vary across studies and experimental systems [86,88,90].

The transition from superficial colonization to tissue invasion may be facilitated by biofilm formation, which shields microorganisms from environmental stressors and sanitizing measures commonly applied during postharvest handling. The molecular resilience of fruit-associated biofilms arises from several overlapping protective mechanisms operating at the cellular and community levels. First, synthesis of a complex exopolysaccharide matrix (glycocalyx) establishes a physical and chemical diffusion barrier capable of sequestering or chemically reacting with antimicrobial agents such as chlorine or hydrogen peroxide, thereby preventing these compounds from reaching embedded microbial cells. Second, the high-density environment within biofilms—frequently encountered during colonization of fruit peels or wounds—induces a general stress response regulated by the alternative sigma factor RpoS (RNA polymerase sigma S). In bacterial model systems and in selected fruit-associated isolates, stress–response regulators such as RpoS have been linked to improved tolerance to oxidative, osmotic, and chemical stress, suggesting a possible role in persistence on fruit surfaces [92,93,94]. Taken together, these mechanisms should be viewed as plausible contributors to persistence on fruit surfaces, but their relative importance under commercial storage conditions remains to be fully established. At the same time, fungal biofilm-associated cells of *Penicillium expansum* and *Neofabraea alba* on apple surfaces display a distinct resistance phenotype driven by coordinated activation of efflux transporters, membrane lipid remodeling, and extracellular matrix-associated protection [95,96,97,98].

Specifically, fungal cells reduce intracellular accumulation of fungicide residues and postharvest sanitizers through upregulation of ABC (ATP-binding cassette) multidrug transporters (notably *PDR5*-type genes) and MFS (major facilitator superfamily) transporters (including *AfuA*). Simultaneously, alterations in ergosterol biosynthesis pathways—mediated by *ERG11* (ergosterol 14-alpha-demethylase), *ERG3* (ergosterol C-5 desaturase), and *ERG1* (squalene synthase)—modify membrane lipid composition and decrease membrane permeability to xenobiotics. These mechanisms have been documented in postharvest fungal biofilms on apples and differ fundamentally from bacterial efflux systems typically described in human pathogens [96,98].

In parallel, biofilm-associated cells may benefit from a physicochemical barrier formed by EPS (extracellular polymeric substances), which restricts diffusion, buffers oxidative stress, and creates localized microenvironments that favor persistence under refrigerated or CA storage. These molecular and structural defenses are particularly critical during long-term storage. While CA storage has been shown to conserve overall microbiome diversity, it may also allow initially limited contamination by pathogens such as *Neofabraea alba* or *Penicillium expansum* to persist and gradually expand over time. This is especially relevant for apples, where extended storage periods may enable low-level contamination to develop into economically significant spoilage. Quantitative PCR (qPCR) analyses have confirmed that even in asymptomatic fruit, the DNA load of pathogens such as *Neofabraea alba* increases significantly over time, reaching peak levels toward the end of the storage season. Consequently, even minor surface damage can have disproportionate effects on fruit quality, shelf life, and consumer safety [92,96,97,98].

From a mechanistic perspective, persistence of *Penicillium expansum* and other spoilage fungi on apple tissues is further supported by transcriptional reprogramming linked to membrane lipid metabolism, ergosterol biosynthesis, organic acid production, autophagy (cellular self-degradation process), MAPK (mitogen-activated protein kinase) signaling, and pectin degradation. In postharvest fungal isolates and apple-infection models, these responses have been associated with enhanced adaptation to the fruit niche and tolerance to host-derived and storage-related stresses; however, these findings come mainly from controlled experimental systems rather than direct in situ observations on commercial fruit. These processes may contribute to adaptation to the fruit niche and to tolerance of host-derived and storage-related stresses, although their relative importance under commercial storage conditions remains incompletely resolved. In postharvest systems, this adaptive capacity is compounded by the decline of fruit defense responses during storage, which is associated with a shift in the microbiome toward spoilage-associated taxa and delayed suppression of fruit rot when defense signaling is experimentally maintained. Understanding the functions of genes contained in the fruit microbiome is crucial for developing new, precise bioprotection strategies (e.g., by designing synthetic microbial consortia) that could extend the storage life of fruit while reducing the use of agrochemicals.

Overall, the susceptibility of fruits to postharvest spoilage reflects the combined effects of tissue composition, microbial ecology, and mechanical integrity of the peel. Because fruits remain living substrates after harvest, their surface microbiota can shift rapidly toward dominance by spoilage-associated organisms under favorable conditions. Biofilm-associated contamination on apple surfaces should therefore be viewed not merely as passive colonization, but as a dynamic survival strategy that increases resilience to chemical control measures and contributes to economically important decay during prolonged storage. These observations underscore the importance of minimizing physical damage, reducing contamination during handling, and maintaining appropriate storage conditions to limit microbial proliferation and preserve fruit quality. Overall, the mechanistic interpretation of fruit-associated persistence should remain tentative, because the supporting data span fruit-surface studies, postharvest assays, and laboratory models, which do not all directly reflect commercial storage environments.

## 6. Consumer Health Risks Associated with Fruit-Borne Pathogens: Selected Molecular Mechanisms of Pathogenicity

The surface of fruits may harbor numerous hazardous pathogens that can affect both the fruit itself and the consumer. Pathogenic bacteria, especially those present in biofilms, pose a threat to consumer health and safety. Studies indicate that microbial cells embedded within a biofilm can be up to 1000 times more resistant to antimicrobial agents compared with planktonic cells. For instance, mature biofilms of *Pseudomonas aeruginosa* and *Staphylococcus epidermidis* exhibit markedly increased tolerance to antibiotics such as tobramycin and ciprofloxacin, which further increases as the biofilm community enters the stationary phase [95]. In the context of fresh produce, enteric pathogens such as *Salmonella enterica* and *Listeria monocytogenes* form persistent biofilms on fruit surfaces and processing lines [92,99]. The extracellular matrix of these biofilms provides a physical barrier that limits the efficacy of standard sanitizers: for example, typical chlorine treatments (50–200 ppm) often achieve only a 1–2 log CFU/g reduction [99]. Therefore, understanding the biofilm present on fruits is crucial for preventing contamination, predicting pathogen growth, and ensuring food safety and quality throughout the supply chain. Among the most frequently isolated bacteria, the predominant strains are *E. coli*, *L. monocytogenes* and *Salmonella* spp. [92,99].

One example of a risk to humans from eating contaminated fruit is the development of listeriosis, a serious infection caused by *L. monocytogenes*. The 2011 multistate outbreak of listeriosis in the United States, linked to contaminated cantaloupes (melon), demonstrated that fresh fruit can serve as a significant vehicle for pathogenic microorganisms [100,101]. The clinical outcome of *L. monocytogenes* infection depends on bacterial load, strain virulence, and host immunity. In immunocompetent individuals, infection is typically asymptomatic or manifests as mild febrile gastroenteritis. Severe invasive listeriosis predominantly affects pregnant women, neonates, older adults, and immunocompromised individuals and may lead to bacteremia, meningitis, meningoencephalitis, or fetal loss. At the molecular level, the pathogenicity of *L. monocytogenes* is driven by a coordinated set of virulence factors that enable intestinal invasion, intracellular survival, and systemic dissemination. The pathogen initiates infection by adhering to and invading intestinal epithelial cells through surface-associated virulence factors, primarily the internalins InlA and InlB, which bind to E-cadherin and c-Met, respectively, thereby facilitating bacterial uptake into host cells. Following internalization, *L. monocytogenes* escapes the phagosome through the action of listeriolysin O and phospholipases, enabling replication within the host cell cytoplasm. The bacterium subsequently spreads from cell to cell via ActA-mediated actin polymerization, promoting intracellular motility and evasion of the host immune response [102]. Expression of these virulence factors is coordinately regulated by the master transcriptional regulator PrfA (27 kDa), encoded within Listeria pathogenicity island 1 (LIPI-1). In response to host environmental cues, particularly physiological temperature (~37 °C), PrfA activates the transcription of key virulence genes while maintaining low expression under environmental conditions [102,103].

In another study, González-López et al. investigated how *Salmonella* attaches to and colonizes three apple varieties: Rayada, Golden Delicious, and Red Delicious [104]. The bacteria were able to form biofilms and survive on the apple surfaces, especially at room temperature. These results indicate that apples can be potential carriers of *Salmonella*, which may form biofilms on the surface of the fruit. Subsequent research published in 2025 by Madad et al. demonstrated that an outbreak of *Salmonella Enteritidis* in the United States and Canada was related to fresh peaches, causing numerous gastrointestinal infections and hospitalizations [105]. These results indicate that foodborne infections caused by this pathogen can arise from contaminated fruits [92,95,99,100,101,104,106]. *Salmonella* spp. primarily causes gastrointestinal infections. Depending on the clinical manifestations, salmonellosis may present as four major disease syndromes: enteric fever (including typhoid and paratyphoid fever), acute gastroenteritis, bacteremia, and other complications of non-typhoidal salmonellosis, including chronic carriage. These infections are particularly severe in young children, older adults, and immunocompromised individuals [106,107]. At the molecular level, the pathogenicity of *Salmonella* varies among species and serovars. *Salmonella enterica*, responsible for the vast majority of human salmonellosis cases, achieves pathogenicity through the coordinated action of virulence determinants involved in adhesion, invasion, and intracellular survival, which are mainly encoded by chromosomal and plasmid genes organized within Salmonella pathogenicity islands (SPIs). Initial attachment to host cells is mediated primarily by fimbrial adhesins, including type 1 fimbriae (Fim), long polar fimbriae (Lpf), and plasmid-encoded fimbriae (PEF), whereas invasion occurs through both the receptor-mediated zipper mechanism and the SPI-1 T3SS-dependent trigger mechanism, each promoting actin cytoskeleton rearrangements and bacterial internalization. After entry, *Salmonella* persists within the Salmonella-containing vacuole (SCV), where SPI-2 T3SS-2 effectors alter intracellular trafficking, inhibit lysosomal degradation, and support intracellular replication and systemic dissemination [107].

Apart from bacteria, fungi and viruses present on fruits represent significant threats to consumer health, as fungi can cause spoilage and produce mycotoxins, while viruses, such as noroviruses and hepatitis A virus, may lead to severe human infections [99,108]. Noroviruses are recognized as one of the principal etiological agents of produce-associated foodborne outbreaks. These non-enveloped, single-stranded RNA viruses exhibit considerable environmental stability, retaining infectivity under a wide range of conditions, including fluctuations in pH, desiccation, and exposure to ultraviolet (UV) radiation. Their epidemiological significance is further enhanced by a very low infectious dose, with fewer than 10 viral particles reported to be sufficient to initiate infection. On the surface of fresh fruits, noroviruses may become associated with biofilm matrices, which can limit the effectiveness of chemical sanitizers, including chlorine-based disinfectants. Moreover, viral particles have been shown to penetrate superficial plant tissues, thereby reducing the efficacy of conventional surface decontamination procedures. Epidemiological evidence indicates that noroviruses account for a substantial proportion of produce-associated outbreaks, representing approximately 59% of reported cases in the United States and 53% in the European Union [92,99,109,110].

The most commonly identified pathogens are presented in Table 3.

Fungi, similarly to bacteria, can form biofilms, whose extracellular matrix increases their resistance and complicates their eradication. These microorganisms present on the peels can cause changes to the product surface, promoting growth and the production of toxic compounds, shortening the shelf life and contaminating neighboring fresh products. Fungi are important postharvest fruit pathogens, mainly because they cause spoilage and, in some species, mycotoxin contamination that may pose consumer health risks. Research published in 2025 by Gal et al. identified fungal isolates from postharvest decayed apples, including species of *Penicillium* [26]. Some isolates, such as *Penicillium expansum*, produced significant levels of mycotoxins, posing a potential health risk to consumers [25,26,27,38]. Saleem et al. identified fungal isolates from postharvest apple peel that predominantly belonged to the genera *Penicillium* and *Talaromyces*, including *P. expansum*, *P. crustosum*, and *T. atroroseus*, all of which are associated with blue mold and mycotoxin production [35]. Other studies from Shebany et al. have shown that *Aspergillus niger* was isolated from contaminated pears (*Pyrus communis*) with some isolates producing ochratoxin A [36]. The clinical outcome of exposure to fruit-associated fungi depends on the fungal species involved, the mycotoxins produced, and host susceptibility. Although exposure is often asymptomatic in healthy individuals, it may lead to allergic reactions, gastrointestinal disturbances, and toxic effects, including immunotoxicity, neurotoxicity, and genotoxicity [26,35,36]. At the molecular level, pathogenic fungi associated with fruit employ diverse virulence mechanisms that vary among species. Nevertheless, several common strategies can be identified, including the secretion of cell wall-degrading enzymes, production of secondary metabolites and, in some cases, mycotoxins, as well as adaptation to host-associated environmental stress. One well-characterized example is the fungal pathogen *P. expansum.* At the molecular level, its pathogenicity relies on the coordinated action of cell wall-degrading enzymes, gluconic acid-mediated tissue acidification, and PacC-dependent pH regulation, which facilitate host tissue colonization and promote patulin biosynthesis. Patulin, a toxic secondary metabolite whose biosynthesis is directed by a dedicated gene cluster, represents the major mycotoxin associated with *P. expansum* contamination of apples, and its production is strongly influenced by environmental conditions and the physiological state of the host tissue [26].

Table 4 presents the most common fungi and the diseases they cause, along with their potential risks to consumers’ health [26,35,36,37,38,72,89].

The pathogens listed in Table 3 are not randomly present on fruit surfaces. Their frequent detection is linked to their ability to form biofilms, the composition and stability of which are regulated at the molecular level. These molecular mechanisms not only facilitate pathogen persistence but also enhance their capacity to survive gastrointestinal passage and establish infection in the consumer. Understanding these molecular mechanisms can help inhibit biofilm formation and reduce pathogen persistence on fruits, and the following section details the key molecular factors involved.

## 7. Biofilm Removal Methods

To effectively minimize the risk of microbial contamination, the source and type of contamination must first be identified. Additionally, microbial diversity, the development stage of biofilm-forming organisms, surface type, and any surface imperfections must be considered to implement appropriate preventive measures. Prevention is considered to be the best strategy for biofilm removal. Traditional methods such as rinsing, chlorination and ultraviolet light are used for this purpose, but recent research has led to progress in the development of new techniques such as irradiation, cold plasma, enzymatic destruction, bactericidal coating, electric and magnetic fields, nanotechnology and bioelectrical approaches [111,112]. These methods are divided into physical, chemical and biological methods. Classic mechanical physical methods, such as scraping and scrubbing, have traditionally been used however they are often limited in precision due to production constraints [24]. More accurate physical techniques, including ultrasonication and irradiation using various types of ionizing (gamma, X-ray, high-energy electron beam, UV-C,B) or non-ionizing (UV-A) radiation, are increasingly preferred. Chemical methods provide a versatile alternative, capable of inactivating both microorganisms and their protective biofilm matrix. Examples include ozone, hydrogen peroxide, and peracetic acid. Biological methods are relatively new but highly effective, utilizing natural mechanisms such as bacteriophages, enzymes and biosurfactants [24,111,112,113]. Examples of physical, chemical, and biological methods, along with their key characteristics, are presented in Table 5.

Depending on the stage of biofilm development, different methods and agents are applied for its control [114]. Furthermore, use of only one of the below-mentioned techniques is often not sufficient to achieve a high level of control over microbiological safety and fruit quality. Combining different methods can achieve higher antimicrobial efficacy and reduce the use of large amounts of chemical compounds, which will avoid the risk of their negative impact on the quality and durability of products and consumer safety. Tango et al. demonstrated that washing fruit with calcium oxide for 3 min, followed by a 3 min ultrasound bath containing fumaric acid and slightly acidic electrolyzed water, significantly reduced bacterial counts compared to the use of individual treatments [115]. Another study demonstrated that the combination of ultrasound with organic acids, particularly lactic and malic acids, significantly enhanced the removal of *Escherichia coli* and *Listeria monocytogenes* biofilms from lettuce leaves, achieving reductions of up to 5.54 and 4.23 log CFU/cm^2^, respectively, with efficacy increasing over treatment time and without adversely affecting the sensory and physicochemical quality of the product, thereby supporting its potential as an effective hurdle technology for fresh produce decontamination [116].

**Table 5:** Methods for the control and removal of biofilms.

Method	Technique	General Description	References
Physical methods	washing	Washing is considered a critical step in maintaining food safety and fruit quality. Studies on this process often yield divergent results; however, all agree that its effectiveness depends on four main variables, known as TACT: Time, Action, Concentration, and Temperature. (a) Mechanical Action: Scrubbing or brushing is crucial for physically disrupting the EPS matrix and separating biofilm cells from the surface. (b) Temperature: Higher cleaning solution temperatures typically improve the removal of organic contaminants and biofilms. (c) Contact Time: Sanitation effectiveness decreases the longer the biofilm remains on the surface before washing (older biofilms are more difficult to remove). Additional factors include the type of fruit and the microorganisms present on its surface. These methods are effective against early-stage biofilms and constitute a preliminary step prior to the application of various disinfection methods, as they involve rinsing fruits with pressurized water to mechanically remove contaminants from their surface. Fresh water rinsing alone can reduce the number of microorganisms and extend shelf life, but it only eliminates a small fraction of pathogenic cells in the biofilm, which is why it is combined with the methods mentioned below.	[13,73,111,117]
	irradiation	Some forms of radiation have the ability to reach pathogens embedded in biofilm structures or internalized within plant tissues. The most commonly studied surface disinfection method is UV-C radiation, which damages the DNA of microorganisms, leading to their inactivation. Low doses of UV-C effectively reduce the number of aerobic mesophilic bacteria and yeasts on products such as apples, melons, pomegranates, and lettuce. However, the main limitation of this method is its low penetration capacity, which means the radiation poorly penetrates the biofilm matrix and does not reach cells hidden deep within its structure or in natural fruit cavities, such as apple calyces. Ionizing radiation, including gamma rays, electron beams, and X-rays, has significantly greater penetrating power and has been approved for use on fresh lettuce and spinach, among other products. This allows for a significant increase in microbiological safety and extension of the shelf life of fresh products without negatively affecting their nutritional value (although some sources indicate the possibility of softening tissues and partial loss of the nutritional value of the fruit).	[13,72,73,111,112]
	ultrasound	A safe, non-toxic and environmentally friendly method for controlling biofilms. High-pressure waves cause acoustic cavitation, releasing a large amount of energy, which leads to cellular stress, disruption of the cell wall and DNA damage through the production of free radicals. The technique allows reaching surfaces that are generally difficult to reach for other cleaning methods, and when combined with other disinfectants, they can further enhance the reduction in pathogen numbers.	[72,73,111,112]
	ultraviolet light	As a result of the use of UV light in the wavelength range of 190–280 nm, the DNA helix is distorted and the replication of cells of exposed microorganisms is disrupted. This method is particularly effective against aerobic mesophilic bacteria and yeasts. Despite the benefits of antimicrobial efficacy and the absence of negative impacts on the nutritional and sensory parameters of products, while also avoiding the generation of toxic residues, this process can penetrate superficially, heating the exposed fruit and causing a shading effect. Studies have shown that the extracellular matrix of biofilms can transmit only one-third or less of the radiation, meaning that microorganisms located in deeper layers of the biofilm or in natural fruit cavities, such as apple calyces, remain protected from light and can survive the process. Polymeric substances within the biofilm act as a physical shield, absorbing radiation and protecting bacterial cells from DNA damage. Due to this barrier, UV irradiation alone is often not sufficient to completely eliminate mature biofilms and requires a multi-level approach (synergy), for example by combining it with ozonation or water, where the fresh product can freely move and rotate during irradiation, which allows UV waves to reach the entire surface of the product, and this allows for much higher bactericidal effectiveness.	[13,72,73]
Chemical methods	ozone	The mechanism of action involves the oxidation of microbial cell membranes, leading to their direct destruction. A key advantage of the process is that ozone quickly decomposes into harmless molecular oxygen, leaving no toxic residue in food. It is used, for example, to inactivate mycotoxin-producing fungi and to degrade their mycotoxins. This technology is also used to extend the shelf life and preserve the quality of apples, cherries, peaches, plums, and grapes, among others, by reducing microbial populations and limiting fruit weight loss. However, it is important to remember that ozone is an unstable gas with a short half-life, requiring its generation directly at the point of use. Incorrect process parameters can lead to undesirable quality changes, such as color deterioration in peaches and carrots or a reduction in vitamin C content in pineapples, bananas, and broccoli. Although ozone is highly effective against initial forms, its effectiveness in removing mature biofilms is clearly limited, which is why ozonation is combined with other techniques to achieve synergistic effects: for example, the simultaneous use of ozone and high-power ultrasound allows for a drastic increase in the reduction of pathogenic cells compared to both methods used separately.	[13,72,73]
	peracetic acid	It is considered one of the most effective oxidizing disinfectants used in the food industry for controlling and removing biofilms, characterized by a broad spectrum of activity against microorganisms, including Gram-positive and Gram-negative bacteria, spores, fungi, and viruses. As a low-toxicity agent, PAA is particularly valued for leaving no harmful residues in food, as it decomposes into oxygen, water, and acetic acid, allowing it to be used even without a final rinse in certain fruit and vegetable sanitation processes. Studies confirm that washing with peracetic acid at appropriate doses does not negatively impact the nutritional parameters or sensory quality of fresh produce, making it an attractive, “green” alternative to traditional disinfection with chlorine compounds.	[13,72,111,118]
	hydrogen peroxide	Considered a safe and eco-friendly oxidizing agent, it has been used in disinfection for over a century due to its exceptionally low toxicity and the fact that it decomposes solely into water and oxygen, leaving no harmful residues in food. Although it has potent antimicrobial activity in planktonic form, its effectiveness against biofilms on fruits and vegetables is limited by bacterial defense mechanisms, particularly the production of enzymes such as catalase and superoxide dismutase, which detoxify hydrogen peroxide. Therefore, to achieve the desired bactericidal effect on biofilm structures, very high concentrations (often above 5%) and extended contact times are necessary. This distinguishes it from peracetic acid, which is more effective at lower doses. Studies on fruits such as cantaloupe have shown that the effectiveness of hydrogen peroxide rinsing decreases dramatically if pathogens (e.g., Salmonella) have had time to form a biofilm on the fruit surface for more than 24 h. In the food industry, hydrogen peroxide is valued mainly for its ability to sterilize packaging materials in aseptic systems and disinfect storage equipment, but in the case of mature biofilms on fresh products, it often requires support from other technologies.	[13,111]
Biological methods	bacteriophages	Considered a promising, natural, and non-thermal alternative or complement to traditional chemical disinfectants used to remove biofilms from food and contact surfaces. Their greatest advantage is their high specificity towards the host bacteria or a group of closely related strains, eliminating only targeted pathogens without disrupting the natural microflora. Furthermore, bacteriophages replicate at the site of infection if host cells are present, increasing their effectiveness. Other advantages include the lack of significant side effects, relatively quick and simple isolation, and low production costs. A key step in bacteriophage action is adsorption to specific receptors found on the bacterial surface, such as lipopolysaccharides, outer membrane proteins, and other cellular structures. However, a significant challenge in eliminating biofilms is the presence of the extracellular polymer matrix (EPS), which acts as a barrier that impedes access to bacterial cells. To overcome this barrier, phages utilize diffusion and produce enzymes that degrade biofilm components, enabling the phages to reach cellular receptors, effectively infect bacteria, and lyse them, leading to destabilization and structural reduction. The effectiveness of this technology can be further enhanced by using genetic engineering methods, which enable the construction of bacteriophages that secrete enzymes that degrade the EPS matrix during the infection cycle. Such modified phages demonstrate higher efficiency in biofilm removal than natural strains. Despite numerous advantages, the use of bacteriophages also presents certain limitations. These include a narrow host range, the potential for bacterial resistance to phages, and the risk of releasing bacterial endotoxins during cell lysis. To mitigate these problems, phage cocktails containing mixtures of different bacteriophage strains and genetically modified phages are used. As a result, they are considered highly effective antibiofilm agents which, after appropriate selection and optimization, can become a standard tool for the safe decontamination of fruits, vegetables and other food products.	[112,113,118]
	edible coatings	Many fruits contain natural wax, which protects them from water loss. As a result of various postharvest practices, fruits are deprived of this layer. The idea of edible coatings was created mainly to increase the shelf life of fruits and protect them from water loss and changes in sensory characteristics, but new proposals aim to extend their application to enhance microbiological safety by adding antimicrobial agents to their composition. The use of edible coatings is an innovative and eco-friendly strategy for protecting fruits and vegetables. They involve creating thin layers on their surfaces that mimic or enhance natural plant waxes. These coatings, made from renewable raw materials such as lipids, proteins, and polysaccharides (including starch, cellulose derivatives, chitosan, pectin, and alginate), act as physical barriers that limit water loss, fruit respiration, and enzymatic oxidation. A key advantage of this technology is its ability to improve microbiological safety by directly inhibiting the growth of pathogens and reducing the number of mesophilic bacteria, yeasts, and molds. Materials such as chitosan exhibit natural antimicrobial activity, effectively limiting the growth of bacteria such as *S. aureus* and coliform bacteria on products such as baby carrots and strawberries. Furthermore, edible coatings have enormous potential as carriers of active ingredients, including antioxidants, colorants, flavors, and natural preservatives such as bacteriocins and essential oils, enabling synergistic biofilm control and extended product shelf life.	[72,73]
	bacteriocins	Ribosome-synthesized antimicrobial peptides, produced by all prokaryotic lineages and active mostly against closely related species. They act as narrow-spectrum antibiotics, binding to a specific component of the cell wall in the target cell, influencing its cycle. Their mechanism of action is multi-level: at an early stage, they inhibit initial cell adhesion to the surface (often by altering its hydrophobicity), while in mature biofilm structures, they lead to cell inactivation by damaging membrane integrity (pore formation, loss of proton-motive force, cytoplasmic leakage), inhibiting peptidoglycan synthesis, and disrupting intracellular processes such as DNA replication and QS communication. In practice, bacteriocins can be applied directly to fruit in pure form, as a component of edible coatings (e.g., chitosan- or alginate-based), or released from active polymer packaging, which provides long-term protection against secondary contamination and extends the shelf life of products without negatively impacting their sensory quality. Despite their great potential, the narrow spectrum of activity of some bacteriocins against multi-species biofilms remains a challenge, prompting researchers to create modified bacteriocin cocktails and utilize genetic engineering to design new generations of bioprocides.	[72,73,112,118]
	essential oils	They exhibit strong antimicrobial and preservative properties, while also exhibiting low mammalian toxicity, rapid environmental degradation, and a low risk of inducing bacterial resistance, making them a safe alternative to synthetic disinfectants. The mechanism of action of essential oils primarily involves damaging the microbial cell membrane and wall, leading to loss of cell integrity and cell death. Furthermore, these compounds inhibit biofilm formation by limiting bacterial motility and interfering with the quorum sensing process, which coordinates the expression of genes involved in biofilm adhesion and development. As a result, they can both prevent the formation of biofilms and facilitate their removal from the surfaces of fruits and vegetables. Among the best-known substances are oregano oil, which effectively prevents the formation of biofilms by staphylococci and Escherichia coli, and facilitates their elimination. Thyme oil, whose main component—thymol—is one of the most effective biofilm inhibitors, demonstrates activity even at very low concentrations. In industrial practice, essential oils can be used in the form of nanoemulsions or aqueous preparations, which increases their stability and antimicrobial effectiveness. Their use in combination with other food preservation methods yields particularly promising results. For example, enriching edible protective coatings, such as chitosan or alginate coatings, with bergamot oils and compounds such as eugenol, thymol, or carvacrol not only effectively inhibits bacterial and fungal growth but also reduces fruit respiration and water loss. Beneficial effects of this approach have been demonstrated in grapes, blueberries, mangoes, and strawberries, among others.	[119,120]

## 8. Discussion and Conclusions: Pesticide Residues, Apple Surface Microstructure, and Biofilm-Mediated Pathogen Persistence

The evidence summarized in this review supports the hypothesis that fruit surfaces are dynamic ecological interfaces rather than passive barriers. The interaction between pesticide residues and surface microstructure represents a novel analytical focal point, explaining why pathogens remain present even after recommended preharvest intervals. Despite the widespread application of pesticides to control phytopathogens and pests, numerous studies demonstrate that these treatments do not fully eliminate human pathogens. Microorganisms such as *Salmonella* spp., *Escherichia coli* O157:H7, *Listeria monocytogenes*, and *Shigella* can survive pesticide applications and remain present on fruit peels even after recommended preharvest intervals, ultimately reaching the consumer [93,94]. However, these observations should be interpreted with caution, as many of the supporting studies were performed under controlled experimental conditions that may not fully reflect the complexity of commercial orchards, postharvest handling, and storage environments. This persistence highlights both the resilience of microbial communities associated with fruit surfaces and the limitations of pesticide-based approaches in ensuring microbiological safety. At the molecular level, this survival is often facilitated by the induction of general stress response regulators, such as the alternative sigma factor RpoS in *Salmonella* and *E. coli*, or the sigma factor rB in *L. monocytogenes*, which orchestrate the expression of genes involved in cross-protection against osmotic and chemical stressors [94]. Furthermore, specific pesticide formulations, like the fungicide chlorothalonil (Bravo 500), have been shown not only to be non-inhibitory but actually to support the survival and growth of these pathogens, likely due to the presence of inert ingredients that serve as nutrient sources or the ability of the bacteria to maintain cytoplasmic homeostasis despite the chemical challenge [93]. Together, these findings indicate that pesticide exposure may reshape, rather than simply suppress, the surface-associated microbial habitat.

A key factor contributing to this phenomenon is the complex microstructure of fruit peels. In apples, for instance, lenticels—microscopic openings that increase in size and number during fruit maturation—serve as natural conduits for gas exchange but also create microenvironments that facilitate pesticide penetration and microbial colonization. These structures may enable the internalization of pathogens beneath the fruit surface, rendering conventional decontamination strategies, such as washing or surface disinfection, largely ineffective [121]. At the same time, the extent to which these microstructural features drive microbial internalization in situ remains insufficiently validated, since much of the available evidence is derived from model systems or indirect observations rather than direct analyses of intact fruit surfaces. This structural complexity underscores a critical gap in understanding the interactions among fruit anatomy, pesticide residues, and microbial ecology. Electron microscopy reveals that the apple cuticle consists of a reticulate-lamellate architecture, where the internal cuticular layer contains a network of tubules composed of polysaccharide microfibrils. These microtubules serve as transport pathways for epicuticular wax molecules and water, but may also act as sub-microscopic channels for the passive infiltration of bacteria. The “tear and repair” mechanism of the wax layer, while intended to prevent water loss, can trap internalized pathogens within microcracks, shielding them from the external environment [121].

Compounding this issue, pesticide residues themselves may inadvertently promote microbial survival and biofilm formation. Certain classes of pesticides, including organophosphorus compounds, carbamates, and pyrethroids, can alter the physicochemical conditions on fruit surfaces, potentially reducing natural microbial competition and the effectiveness of sanitizing agents. For example, a field study conducted in a Spanish vineyard (cv. Tempranillo) demonstrated that while biological fungicides based on *Bacillus subtilis* QST713 maintained the ecological balance of the grape surface, chemical treatments with fenhexamid significantly reduced microbial community richness and diversity. Interestingly, in this specific experimental context—characterized by an absence of initial *Botrytis cinerea* infection—the chemical fungicide was associated with the subsequent detection of *B. cinerea* DNA in grape biofilms, a pathogen not found in the control or bio-treated samples [6]. Further evidence from greenhouse experiments on pepper plants (*Capsicum annum*) highlights the role of systemic pesticides in altering phyllosphere dynamics. Foliar application of the insecticide imidacloprid was shown to significantly shift fungal communities, specifically stimulating the growth of basidiomycetous yeasts (*Cryptococcus* spp.), potentially as a metabolic response to the chemical stressor. Similarly, the systemic fungicide metalaxyl was found to stimulate specific *Enterobacteriaceae* and the fungus *Periconia macrospinosa*. These findings suggest that pesticide residues can selectively promote certain taxa, potentially aiding the persistence of opportunistic or resistant microorganisms within the biofilm matrix [7]. Thus, the main pattern emerging across fruit and phyllosphere systems is not uniform suppression, but selective ecological reshaping. While these studies provide strong molecular evidence of microbial shifts, several limitations must be considered. First, the data from pepper plants were obtained in a controlled greenhouse setting, which may not fully replicate the extreme environmental fluctuations (UV radiation, temperature, and desiccation) found in open-field orchards. Second, much of the evidence relies on PCR-DGGE and cloning techniques, which are subject to inherent limitations such as the detection of DNA from non-viable cells and the potential for diverse sequences to co-migrate as a single band, which may underestimate true taxonomic complexity. While recent high throughput metagenomic studies have begun to provide higher resolution, the transition between these different datasets introduces variability in how “core” microbiomes are defined across the literature [15,74].

The formation of biofilms is particularly problematic, as microorganisms embedded within these structured communities exhibit increased resistance to chemical treatments and mechanical removal. Moreover, biofilms enhance microbial stability and metabolic cooperation, contributing not only to persistent contamination but also to fruit spoilage through enzymatic degradation and the development of off-flavors. The molecular basis for this enhanced resilience involves QS, where plant-associated bacteria produce signaling molecules such as acyl-homoserine lactones (AHLs) that may upregulate stress-resistance factors in immigrant pathogens. Additionally, the presence of pathogens can trigger the Type III Secretion System (TTSS), a conserved apparatus among Gram-negative bacteria used to deliver effector proteins into host cells, which may also play a role in the stable attachment and colonization of the fruit biome [94]. However, the relevance of these processes should be interpreted cautiously, because much of the supporting evidence derives from laboratory models, planktonic cultures, or non-fruit isolates rather than direct observations on fruit surfaces under commercial storage conditions.

The coexistence of pesticide residues and pathogenic biofilms therefore represents a compounded risk to consumer health and product quality. Studies indicate that fruits produced under conventional agricultural systems often contain higher levels of both pesticide residues and microbial contaminants compared to those from organic or integrated pest management systems, likely due to intensive chemical use and potential cross-contamination during handling [122]. Additionally, pesticide residues may accumulate in the fruit peel and, in some cases, penetrate into the pulp via lenticels or microcracks, further complicating removal. Standard household washing methods, including rinsing with water or mild detergents, are frequently insufficient to eliminate either chemical residues or biofilm-associated microorganisms [123,124,125,126]. Chemical mitigation often requires advanced oxidation processes; for instance, sonolysis via ultrasonication can degrade organophosphorus pesticides like phorate or diazinon by generating hydroxyl radicals (•OH) through acoustic cavitation [126]. These highly reactive radicals attack specific molecular sites, such as P=S and C-S-C bonds, mineralizing toxic residues into less harmful compounds like CO_2_ and H_2_O. However, without such targeted molecular degradation, residues of lipophilic pesticides tend to partition into the waxy cuticle layers, effectively sequestering both the chemical and any associated microbial biofilms from conventional surface decontamination [125].

Taken together, these findings are compelling but still incomplete. A more cautious interpretation is warranted because the current literature rarely integrates chemical residue dynamics, microbial viability, and fruit surface ultrastructure within a single standardized framework. Future studies should therefore rely more consistently on field-based designs and harmonized analytical methods to improve comparability and ecological relevance. These challenges highlight the necessity for advanced monitoring and intervention strategies that address both chemical and microbiological hazards simultaneously. In particular, greater attention should be paid to biofilms as dynamic systems that respond to pesticide exposure, potentially altering microbial community composition, resistance, and pathogenicity. On a molecular level, the exopolymeric matrix of these biofilms acts as a protective shield, sequestering xenobiotics and inducing the general stress response. This regulatory activation facilitates cross-protection mechanisms, where exposure to sublethal concentrations of pesticides can paradoxically enhance the resilience of immigrant pathogens to subsequent environmental stressors, such as desiccation, UV-B radiation, or osmotic shifts [94]. Understanding how pesticides influence these dynamics could provide critical insights for improving postharvest sanitation and reducing foodborne risks. To mitigate these issues, integrated approaches combining optimized pesticide use, improved hygienic practices during harvesting and processing, and targeted postharvest treatments are essential. Emerging technologies, such as enzymatic biofilm disruption, biocontrol agents, and advanced oxidation processes, offer promising avenues for enhancing the removal of both pesticide residues and microbial contaminants. The implementation of sonolysis via high-power ultrasound represents a key advanced oxidation strategy; acoustic cavitation generates localized “hot spots” with extreme temperatures (5000 K) and pressures (1000 atm), facilitating the production of non-selective hydroxyl radicals that mineralize toxic residues. Additionally, the targeted application of enzymes, such as organophosphorus hydrolases or laccases, allows for the precise biotransformation of contaminants into non-toxic metabolites through enzymatic cleavage [122,125,126].

Additionally, consumer education on effective washing techniques, including the use of acidic or alkaline solutions, may further reduce exposure risks. The efficacy of these household treatments is largely governed by the physicochemical properties of the residues, specifically their water solubility and octanol–water partition coefficient (Log *K_ow_*); for instance, highly lipophilic pesticides that partition into the waxy cuticle layers often require specific pH adjustments or the use of vinegar-based solutions to be successfully dislodged [125]. Long-term solutions may also involve breeding and biotechnological strategies aimed at modifying fruit surface properties. Reducing lenticel density or strengthening cuticular barriers could limit both pesticide penetration and microbial ingress. Ultrastructural modifications targeting the reticulate-lamellate architecture of the cuticle or optimizing the “tear and repair” (rip and stitch) mechanism of epicuticular waxes could significantly decrease the infiltration of pathogens through sub-microscopic polysaccharide microtubule networks [121]. Complementary implementation of Good Agricultural Practices (GAPs), including reduced pesticide application rates and improved supply chain hygiene, is equally important for minimizing contamination at the source. In this context, the integration of Artificial Intelligence (AI) and Machine Learning (ML) offers a transformative approach, utilizing predictive modeling systems like dynamiCROP to optimize preharvest intervals and AI-assisted hyperspectral screening to ensure compliance with stringent maximum residue limits (MRLs) through real-time data management across the supply chain [125]. Overall, the evidence indicates that effective risk management requires coordinated chemical, microbiological, and structural assessment, alongside field-based validation of mitigation strategies.

## 9. Future Research Perspectives

From a research perspective, future work should prioritize integrated studies that combine fruit anatomy, surface chemistry, and microbiology, as this review demonstrates that these factors jointly determine safety outcomes during prolonged storage. Priority should be given to identifying how specific fruit surface structures influence the penetration of pesticide residues and the establishment of biofilms, as well as how different microbial communities respond to repeated chemical exposure. In particular, investigating the reticulate-lamellate architecture of the cuticle is essential, as the network of tubules composed of polysaccharide microfibrils may act as sub-microscopic channels for the passive infiltration of bacteria into internal tissues [94]. These structural features have already been explored in fruit cuticle research, and future studies should therefore focus on linking them more directly to microbial persistence and residue behavior under realistic postharvest conditions. High-resolution imaging, metagenomics, transcriptomics, and metabolomics should be used together to define the mechanisms governing persistence, resistance, and pathogenicity on fruit surfaces. Metagenomic profiling could elucidate how immigrant pathogens compete with native epiphytic microflora for carbon sources, while transcriptomics can track the expression of general stress response regulators, which orchestrate cross-protection against subsequent environmental challenges. Such approaches would help distinguish transient contamination from stable colonization and identify the conditions under which biofilms become most difficult to remove.

Development efforts should also focus on practical interventions that reduce risk across the entire supply chain. These should include optimized pesticide application, improved orchard hygiene, gentler harvesting and sorting practices, and targeted postharvest decontamination methods designed specifically to disrupt biofilms. Several of these approaches, including cuticle modification and breeding-related surface traits, are already being actively investigated and should be treated here as areas requiring validation and refinement rather than as entirely new proposals. Future strategies must leverage synergistic “hurdle technologies,” such as combining sonolysis with enzymatic treatments. Another important direction is the development of monitoring systems capable of detecting both chemical and microbiological hazards in a single framework. This would allow risk assessment to move beyond isolated residue testing toward a more realistic evaluation of the fruit surface as a living and chemically dynamic habitat. Such an approach would be especially valuable for long-storage fruits, where even low-level contamination may persist and amplify over time. Consumer-level guidance may also remain useful, but it should be treated as a secondary barrier rather than a primary safeguard. In summary, protecting consumers from fruit biofilms will require a multidisciplinary strategy that combines crop protection, postharvest hygiene, surface microbiology, and innovative decontamination technologies. The most effective future research should focus on the fruit surface as an integrated system, where structural vulnerability, pesticide exposure, and microbial ecology jointly determine safety outcomes. This perspective should be framed as an extension of ongoing work on fruit cuticle biology rather than as an unexplored research direction. Molecular insights into quorum sensing—where signaling molecules like acyl-homoserine lactones (AHLs) trigger the stable colonization of pathogens—along with the role of the Type III Secretion System (TTSS) in attachment, will be pivotal for designing next-generation biocontrol agents [94]. These pathways have already been studied in related plant-associated and food microbiology systems, but their specific contribution to fruit-surface persistence remains insufficiently resolved. Only by addressing these factors together can the food chain be made more resilient and the risks connected with biofilm-associated contamination be substantially reduced [121,122,123,124,125,126].

## Figures and Tables

**Table 1:** Representative epiphytic bacteria commonly found on fruit surfaces. Gram-negative and Gram-positive bacteria are presented separately with their taxonomic classification from phylum to genus.

Funcional Category	Phylum	Class	Order and Family	Genus	Literature
Resident epiphytes	Pseudomonadota	Gammaproteobacteria	Pseudomonadales → Pseudomonadaceae	*Pseudomonas*	[16,17,19]
		Alphaproteobacteria	Sphingomonadales → Sphingomonadaceae	*Sphingomonas*	[16,17,19]
			Rhizobiales → Methylobacteriaceae	*Methylobacterium*	[16,17,19]
	Bacteroidota	Flavobacteriia	Flavobacteriales → Flavobacteriaceae	*Flavobacterium*	[17,19]
	Actinomycetota	Actinobacteria	Micrococcales → Micrococcaceae	*Micrococcus*	[17,19]
			Micrococcales → Microbacteriaceae	*Microbacterium*	[17,19]
Spoilage organisms	Pseudomonadota	Gammaproteobacteria	Enterobacterales → Enterobacteriaceae	*Enterobacter*, *Enterobacter*, *Escherichia*, *Citrobacter*	[17,44,80]
			Enterobacterales → Erwiniaceae	*Erwinia*	[17,44]
	Bacillota	Bacilli	Bacillales → Bacillaceae	*Bacillus*	[17,44]
			Lactobacillales → Streptococcaceae	*Streptococcus*	[44]
			Lactobacillales → Lactobacillaceae	*Lactobacillus*	[44]
Plant pathogens	Pseudomonadota	Gammaproteobacteria	Enterobacterales → Erwiniaceae	*Erwinia*	[20,44]
			Xanthomonadales → Xanthomonadaceae	*Xanthomonas*	[20,80]
Human pathogens	Pseudomonadota	Gammaproteobacteria	Enterobacterales → Enterobacteriaceae	*Enterobacter*, *Escherichia*, *Citrobacter*	[17,80]
			Pseudomonadales → Pseudomonadaceae	*Pseudomonas*	[80]
	Bacillota	Bacilli	Bacillales → Staphylococcaceae	*Staphylococcus*	[80]
		Clostridia	Clostridiales → Clostridiaceae	*Sarcina*	[80]

**Table 2:** Representative filamentous fungi (molds) and yeasts commonly found on fruit surfaces. Filamentous fungi are separated from yeasts, with their taxonomic classification presented from phylum to genus.

Funcional Category	Phylum	Class	Order and Family	Genus	Literature
Resident epiphytes	Ascomycota	Dothideomycetes	Dothideales → Aureobasidiaceae	*Aureobasidium*	[15,39]
			Capnodiales → Cladosporiaceae	*Cladosporium*	[15,39]
			Capnodiales → Mycosphaerellaceae	*Ramularia*	[15,20]
	Basidiomycota	Exobasidiomycetes	Exobasidiales → Brachybasidiaceae;	*Golubevia*	[15,20]
		Wallemiomycetes	Wallemiales → Wallemiaceae	*Wallemia*	[39,41]
Spoilage organisms	Ascomycota	Eurotiomycetes	Eurotiales → Aspergillaceae	*Penicillium*, *Aspergillus*	[39,44]
	Mucoromycota	Mucoromycetes	Mucorales → Rhizopodaceae	*Rhizopus*	[44]
	Ascomycota	Saccharomycetes	Saccharomycetales → Saccharomycetaceae	*Saccharomyces*, *Zygosaccharomyces*	[44]
			Saccharomycetales → Saccharomycodaceae	*Hanseniaspora*	[44]
			Saccharomycetales → Debaryomycetaceae	*Debaryomyces*	[44]
			Saccharomycetales → Pichiaceae	*Pichia*	[44]
Plant pathogens	Ascomycota	Dothideomycetes	Pleosporales → Pleosporaceae	*Alternaria*	[15,20]
			Pleosporales → Didymosphaeriaceae	*Paraconiothyrium*	[15,20]

**Table 3:** Most frequently detected pathogens on fruit surfaces [72,88,89,92,99,109,110].

Pathogens	Genus/Species	Biofilm Formation Potential	Food Safety Relevance
Bacteria	*Bacillus cereus*, *Campylobacter* spp. (selected strains), *Clostridium botulinum*, *Enterobacter*, *Listeria monocytogenes*, *Salmonella* spp. (selected strains), *Shiga toxin–producing Escherichia coli* (including *E. coli O157:H7*), *Shigella* spp. (selected strains), *Pseudomonas* spp., *Yersinia enterocolitica*	Biofilm formation reported for selected bacterial species/strains.	Major foodborne pathogens; frequent cause of outbreaks linked to fresh fruits.
Fungi	*Penicillium* (mainly *P. expansum*), *Aspergillus* (mainly *A. niger*), *Alternaria* spp., *Fusarium* spp. (selected strains), *Rhizopus* spp. (selected strains)	Biofilm-like structures or surface colonization reported in some species.	Postharvest fruit decay and mycotoxin contamination.
Viruses	noroviruses and others (e.g., hepatitis A virus, adenoviruses and rotaviruses)	Do not form biofilms; may be associated with microbial biofilm matrices.	Human pathogens capable of causing foodborne infections.

**Table 4:** Selected fruit-associated fungal pathogens, associated diseases, and potential health risks by fruit type [26,35,36,37,38,72,89].

Type of Fungal Agent	Common Fruit	Disease Caused	Consumer Health Risk
*Aspergillus* (mainly *Aspergillus niger*)	apples, pears, grapes	Black mold	can produce ochratoxin A; may cause allergic reactions
*Penicillium expansum*	apples, pears	Blue rot or blue mold	can produce mycotoxins lead to gastrointestinal disturbances, immunotoxicity, neurotoxicity and genotoxicity
*Rhizopus* spp.	peaches, plums	Soft rot	can cause mucormycosis in immunocompromised individuals (opportunistic infection)

## Data Availability

No new data were created or analyzed in this study. Data sharing is not applicable to this article.
